# End-of-life prognostic models in advanced cancer: a scoping review of model development, validation, and impact

**DOI:** 10.1186/s12904-026-02172-3

**Published:** 2026-07-03

**Authors:** Caitlin Medlock, Bella Vivat, Alaz Bicaco, Andrea Bruun, Nicola White

**Affiliations:** 1 Cicely Saunders Institute of Palliative Care, Policy & Rehabilitation, Florence Nightingale Faculty of Nursing, Midwifery & Palliative Care, King’s College London, London, United Kingdom; 2 Marie Curie Palliative Care Research Department, Division of Psychiatry, University College London, London, United Kingdom; 3 Clinical Mental Health Sciences, Division of Psychiatry, University College London, London, United Kingdom; 4 Department of Public Health, Children‘s, Learning Disability and Mental Health Nursing, Kingston University London, London, United Kingdom

**Keywords:** Advanced cancer, Prognosis, Scoping review, Survival analysis, Prediction model

## Abstract

**Background:**

Accurate survival estimation is important for clinical decision-making in advanced cancer. Prognostic models can support prediction of end-of-life and should ideally undergo staged evaluation across a development framework encompassing model development, validation, and impact evaluation before clinical implementation. No synthesis currently maps existing models across this framework, making it difficult to determine which tools are available and the extent to which they are ready for clinical use. This review aimed to identify end-of-life prediction models for advanced cancer and map them across this development framework.

**Methods:**

This scoping review included studies reporting prognostic models combining at least two predictor variables to predict end-of-life (≤ 12 months) in adults with advanced cancer. MEDLINE, Embase, CINAHL, and the Cochrane Library were searched from inception to August 2025. Studies were classified as model development, internal validation, external validation, or impact evaluation, with findings summarised descriptively and narratively.

**Results:**

A total of 106 studies were included: 17 (16%) reported development only, 36 (34%) internal validation, and 53 (50%) external validation. Across all studies, 77 distinct models were identified: 8 (10%) had undergone development only, 34 (44%) had been internally validated, and 35 (46%) externally validated. However, most externally validated models had been evaluated only once and within a single country, limiting confidence in their generalisability. No models had undergone impact evaluation.

**Conclusions:**

Although many end-of-life prognostic models exist for advanced cancer, fewer than half have been externally validated and none have been assessed for clinical impact. Their validity, generalisability, and usefulness therefore remain uncertain. Future research should prioritise independent external validation and robust impact evaluation before routine clinical implementation.

**Supplementary Information:**

The online version contains supplementary material available at https://doi.org/10.1186/s12904-026-02172-3.

## Background

### Rationale

The final year of life for people living with advanced cancer is often associated with major decisions about treatment, care, family matters, and personal priorities [1]. Patients may, for example, consider whether to continue disease-directed therapies or instead prioritise symptom management, psychosocial support, and comfort-focused care [2, 3]. In this context, an informed estimate of survival is central to timely and appropriate decision-making [4].

However, prognostication in advanced cancer is inherently difficult. In clinical practice, survival estimates are most commonly based on clinician judgement, referred to as Clinician Prediction of Survival (CPS), however, research has consistently demonstrated that CPS is often inaccurate and overly optimistic [5]. Multiple prognostic models have been developed to support prognostication in advanced cancer [4]. These models typically combine two or more variables to generate a multivariable tool that may provide more objective and reproducible estimates than CPS alone, with the potential to reduce prognostic uncertainty and support prognosis-based decision-making for patients with advanced cancer [6, 7].

The PROGnosis Research Strategy (PROGRESS) framework outlines areas where the design, conducts, and reporting of prognosis research studies can be improved [7–10].

Within this framework, they describe three key steps to making prognostic models useful: (1) development; (2) validation; and (3) impact evaluation. Model development studies aim to derive a prognostic model by selecting the relevant predictors and combining them statistically into a multivariable model [7]. Prognostic models should then undergo some form of validation. Internal validation uses the original study sample, to quantify any optimisms in the predictive performance (e.g., calibration and discrimination) of the developed model [11]. It is also strongly recommended to externally validate developed models, to evaluate the performance of the model in other participant data, either from participants sampled from a later period (temporal validation) or from another setting or country (geographical validation) than what was used for the model development [12, 13].

Impact evaluations are required to establish whether use of the model changes clinical decision-making or patient outcomes in the intended way, and therefore whether it has real clinical value [7, 13]. Only an impact evaluation can determine whether use of a prognostic model is better than usual care [14]. These steps (i.e., development, validation and impact evaluation) are hereafter referred to as the “prognostic model development framework”.

Previous systematic reviews have identified and summarised prognostic models developed for predicting end-of-life in advanced cancer, with a primary focus on identifying those that have undergone external validation and comparing their predictive performance and accuracy as a way of judging their potential usefulness in clinical practice [15, 16]. However, no contemporary synthesis has mapped these models across the prognostic model development framework. This is important because the value of a prognostic model is determined not only by measures of statistical performance, but also by where it sits along the pathway from development to implementation, the populations and settings in which it has been evaluated, the predictors it uses, and the time horizons it targets. Examining these characteristics can help clarify how models differ, identify where evidence is concentrated, and reveal important gaps in development, validation, and implementation.

### Objectives

The aim of this scoping review was to summarise existing end-of-life prognostic models in advanced cancer and map them across the prognostic model development framework [13, 17].

The review question was: “What prognostic models for predicting end-of-life in adults with advanced cancer have been developed and/or validated and/or evaluated for their impact on clinical decision-making and/or patient outcomes?”

The objectives were to:

1. Identify studies reporting end-of-life prognostic models in advanced cancer.
2. Describe the key characteristics of identified end-of-life prognostic models for advanced cancer.
3. Map the identified end-of-life prognostic models for advanced cancer across the prognostic model development framework.

## Methods

A scoping review was considered the most appropriate methodological approach because the aim was to map, describe, and synthesise the breadth of available evidence, and to identify gaps in the literature. It was not intended to undertake formal quality appraisal, assess risk of bias, or compare the performance of individual models, as these questions have already been examined in existing systematic reviews [15, 16].

### Protocol and registration

This scoping review was conducted in accordance with the JBI methodology for scoping reviews [18] and is reported following the Preferred Reporting Items for Systematic Reviews and Meta-Analyses extension for Scoping Reviews guidelines (PRISMA-ScR) [19]. The review protocol was prospectively registered with the Open Science Framework (OSF) on 7th May 2025 (https://osf.io/wnfge).

### Eligibility criteria

Table 1 illustrates the eligibility criteria applied during the screening process.

**Table 1:** Eligibility criteria

PEOS criteria	Inclusion criteria	Exclusion criteria
Population	Adults (≥ 18 years) with advanced cancer — defined as metastatic, locally advanced, or recurrent and considered incurable. Alternative terms include palliative, terminal, end-of-life, or non-curative.	Populations receiving cancer-directed treatment with curative or disease-modifying intent (e.g. surgery, radical radiotherapy, or systemic anticancer therapy), unless explicitly described as palliative or supportive.
Exposure	Prognostic models, algorithms, tools, or estimates that combine two or more variables to predict end-of-life.	Studies investigating multiple prognostic factors without combining them into a multivariable survival prediction.
Outcomes	End-of-life prediction defined as the last 12 months (or one year) of life [20, 21].	Outcomes other than end-of-life (e.g. progression-free survival, disease recurrence, treatment response, or treatment selection).
Study types	Studies reporting original quantitative data on prediction model development, internal validation, external validation, impact evaluation, or any combination. Study designs followed the TRIPOD statement classification system [22]. Impact evaluation studies were defined as assessment of whether model use influenced clinical decision-making and/or patient outcomes, including decision-analytic models (e.g. Markov) and empirical designs (e.g. cluster randomised trials, before-and-after studies) [14].	Studies conducted exclusively in intensive care units (ICUs), emergency departments, or other acute care settings, where prognostication is typically short-term and context specific.

### Information sources

MEDLINE, Embase, the Cumulative Index to Nursing and Allied Health Literature (CINAHL) Plus, and the Cochrane Library were searched from inception to August 2025. Grey Literature Report (www.greylit.org) and OpenGrey (www.opengrey.eu) were also searched to identify further potentially eligible studies. Reference lists of included studies and relevant reviews were searched to identify additional records (backward citation searching). Forward citation searching was also conducted using Google Scholar to identify studies that had cited the included studies.

### Search

The search strategy comprised four conceptual domains: (1) advanced cancer; (2) end-of-life predictions; (3) prognostic models; and (4) statistical terms relevant to development, validation, and impact evaluation. Synonyms and related terms were identified for each domain from relevant systematic reviews [15, 23–26], and search strings were adapted to the syntax and controlled vocabularies of each database. Searches were limited to human adults and English-language publications owing to resource constraints. No date limit was applied to capture the breadth of the literature. The search strategy is provided in Additional File 1.

### Selection of sources of evidence

Search results were uploaded into *Rayyan* (https://www.rayyan.ai/) and de-duplicated. Titles and abstracts were screened in parallel by three reviewers, with each record independently assessed by at least two reviewers. Articles deemed potentially eligible were subsequently assessed in full text by three reviewers, with each paper independently reviewed by at least two reviewers. Any discrepancies were resolved by consensus within the study team.

### Data charting process and data items

Data extraction was undertaken during full-text screening for all studies deemed eligible for inclusion. A standardised data extraction form was developed by CM using an *Excel* spreadsheet and piloted with the first five included studies; any necessary modifications were discussed and agreed by the study team before full extraction commenced. Extracted items included publication characteristics (i.e., authors, year of publication, country of origin), study characteristics (i.e., design, clinical setting, population, sample size), and prognostic model characteristics (i.e., model name, predictor variables, intended population and setting, and model output/time horizon). Data extracted for each study also included information relevant to the prognostic model development framework (i.e., development, internal validation, external validation, and impact assessment), determined using the TRIPOD statement classification system, which defines five mutually exclusive study types based on the data available for model development and validation [22]. Where a study reported more than one stage of the framework (e.g., both internal and external validation), it was classified according to the highest level of validation achieved, as the TRIPOD typology does not provide a category for studies spanning multiple validation types.

### Synthesis of results

Included studies and identified prognostic models were classified according to the prognostic model development framework. Findings were summarised descriptively using tables and figures, with counts and percentages (n, %) used to report the number of studies and models at each stage of development, validation, and impact evaluation. A narrative synthesis was undertaken to contextualise the tabulated results. In keeping with scoping review methodology, no formal quality appraisal of individual studies was conducted [27].

## Results

### Selection of sources of evidence

**Fig. 1:**
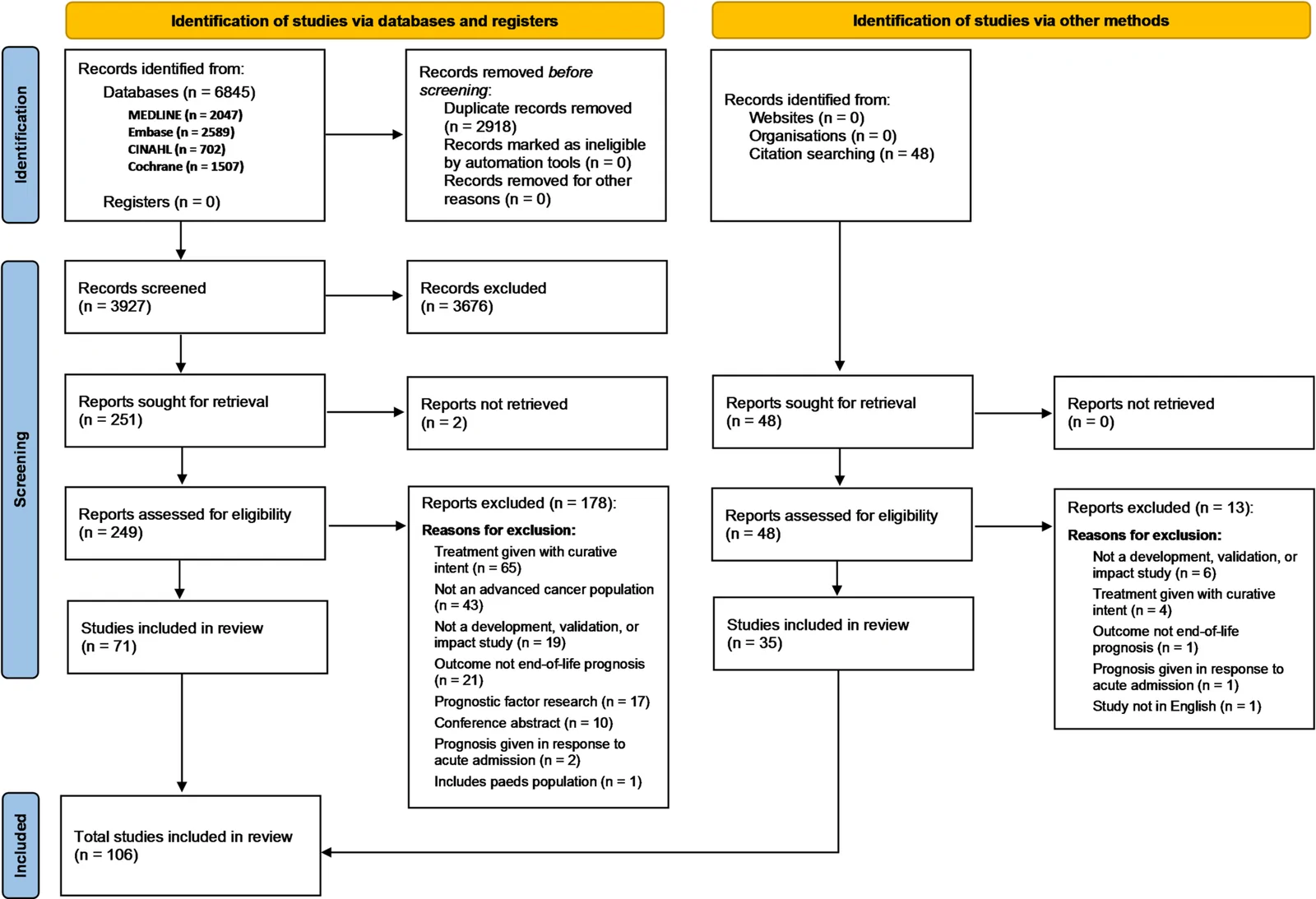
Preferred reporting items for systematic reviews and meta-analyses flow chart

The database search identified 6,845 records. After removal of duplicates, 3,927 records were screened by title and abstract, with 249 full-text articles assessed for eligibility. An additional 35 eligible articles were identified through backwards and forwards citation searching. In total, 106 studies were included in this scoping review. The study selection process is shown in Fig. 1.

### Characteristics of sources of evidence (objective 1)

The cumulative characteristics of included studies are summarised in Table 2. A full list of included studies and their characteristics is summarised in Additional File 2.

**Table 2:** Characteristics of included studies (*n* = 106)

Characteristic	*n*	% ^a^
Study design		
Prospective cohort	55	52
Retrospective cohort	45	42
Mixed (prospective data, retrospective analysis)	4	4
Secondary analysis of RCT	2	2
Continent of study		
Asia	48	45
Europe	27	25
North America	14	13
South America	4	4
Africa & Middle East	4	4
Oceania	2	2
Multi-country collaborations	7	7
Cancer population		
Mixed or heterogeneous cancer populations	85	80
Disease-specific (single cancer type or site)	18	17
Haematological malignancies	3	3
Clinical setting ^b^		
Inpatient (e.g., hospital, PCU, hospice ward, or tertiary cancer centre)	77	73
Outpatient (e.g., cancer centre clinic or palliative radiotherapy department)	40	38
Community or home-based	10	9
Not reported	4	4
Prognostic model development framework classification		
Development studies	17	16
Internal validation studies	36	34
External validation studies	53	50
Impact evaluation studies	0	0
Treatment status of study population		
No ongoing anticancer treatment	34	32
Palliative treatments (e.g., radiotherapy, chemotherapy, TKI, monoclonal antibodies, hormone therapy, or surgery)	45	43
Not reported	27	25

*PCU* Palliative Care Unit, *RCT* Randomised controlled trial, *TKI* Tyrosine kinase inhibitor

^a^ Percentages may not sum to 100% due to rounding

^b^Studies conducted across multiple settings are counted in each applicable category; percentages therefore exceed 100%

Studies were published between 1994 and 2025. Studies were conducted in 31 countries or multinational collaborations, with the largest proportion conducted in Japan (*n* = 22, 21%).

Studies from low- and middle-income countries (LMICs) were underrepresented, with only eleven studies from Brazil (*n* = 5, 5%), Kuwait, Egypt, Saudi Arabia, Indonesia, India, and Thailand (each *n* = 1, 1%) combined. Study designs comprised prospective cohort studies (*n* = 55, 52%), retrospective cohort studies (*n* = 45, 42%), mixed design in which data were collected prospectively but analysed retrospectively (*n* = 4, 4%), and two studies (2%) were secondary analyses of previously conducted randomised controlled trials (RCTs). Sample sizes ranged from 36 to 3,163 participants.

The majority of studies (*n* = 85, 80%) were conducted in mixed or heterogeneous cancer populations rather than disease-specific cohorts. Disease-specific studies (*n* = 18, 17%) included bone metastases, hepatocellular carcinoma, metastatic colorectal cancer, brain metastases, and lung cancer. Three studies (3%) focused on patients with haematological malignancies.

Studies were conducted across a wide range of clinical settings. Inpatient settings, including hospitals, cancer centres, dedicated palliative care units, and hospice wards, were the most common, featuring in 77 studies (73%). Outpatient settings, including cancer centre clinics and palliative radiotherapy departments, were represented in 40 studies (38%). Community or home-based settings featured in 10 studies (9%). Four studies (4%) did not report the clinical setting clearly.

The treatment status of participants varied across studies; 34 studies (32%) included populations no longer receiving anticancer treatments. Patients were receiving palliative treatments in 45 studies (43%), including radiotherapy, chemotherapy, tyrosine kinase inhibitors, and monoclonal antibodies, hormone therapy, and surgery. Twenty-seven studies (25%) did not report patients’ treatment status.

Using the prognostic model development framework, included studies were classified as model development, internal validation, external validation, or impact evaluation studies [13, 17]. Seventeen studies (16%) reported model development only [28–44]; 36 studies (34%) reported internal validation [45–79]; and 53 (50%) studies reported external validation [80–131]. No impact evaluation studies were identified.

### Characteristics of prediction models (objective 2)

A total of 77 end-of-life prognostic models were identified across the 106 included studies. A full summary of the characteristics of the included models can be found in Additional File 3.

The majority of models (*n* = 57, 74%) were designed for patients with advanced solid tumours across mixed primary sites. A minority targeted specific tumour types: bone metastases (*n* = 5, 7%), gastrointestinal malignancies (*n* = 3, 4%), gastric cancer (*n* = 3, 4%), and lung or respiratory malignancies (*n* = 3, 4%). Single models were developed for patients with hepatocellular carcinoma, pancreatic cancer, lung cancer with bone metastases, malignant biliary obstruction, and brain metastases. One model (1%) was developed specifically for haematological malignancies, while two models (3%) were validated or specifically adapted for use in this population.

Most models (*n* = 42, 55%) combined clinical and laboratory variables (e.g. performance status, symptom scores, and oral intake combined with c-reactive protein (CRP), albumin, and white cell count). Nine (12%) relied solely on laboratory parameters (e.g. CRP, albumin, and bilirubin), whereas 26 (34%) used clinical variables alone and did not require blood tests (e.g. performance status, symptom burden, and metastatic site).

The most common approach to prediction was a numerical score stratifying patients into discrete risk groups, used by 37 models (48%). Twenty-three models (30%) produced a continuous survival probability, ranging from simple probability estimates to nomogram-based outputs generating individualised predictions across multiple time points simultaneously. Eleven models (14%) used a binary classification, dichotomising patients into high/low risk or alive/dead categories. Four models (5%) used a decision tree approach, generating risk groups with associated mortality probabilities across discrete nodes. Two models (3%), PiPS-A and PiPS-B, were unique in generating a categorical verbal output, classifying patients as “days”, “weeks”, or “months+”, explicitly designed to align with clinical communication. Time horizons ranged from next-day survival to open-ended overall survival, highlighting variation in how the end-of-life was conceptualised across models. Thirty-day mortality was the most commonly represented time horizon, included in 24 models (31%) either as the sole endpoint or alongside other prediction time points in multi-horizon models.

### Mapping models across the prognostic model development framework (objective 3)

Each identified end-of-life prognostic model was classified using the prognostic model development framework as development only, internally validated, or externally validated [13, 17]. As no impact assessment studies were identified, none of the models had undergone impact evaluation. Table 3 maps the identified models across the prognostic model development framework.

**Table 3:** End-of-life prediction models for advanced cancer (*n* = 77) mapped across the prognostic model development framework

Model Name	Development	Internal Validation			External Validation	
	Development Only	Development + Internal Validation (resampling)	Development + Internal Validation (random split-sample)	Development + Internal Validation (non-random split sample)	Development + External Validation (in same study)	External Validation Only
2-Month Prognostic Score (Barbot Score)	**✓**					
7-Day Survival CART Model / 30-Day Survival CART Model		**✓**				
Artificial Neural Network 30-Day Survival Model		**✓**				
Barnholtz-Sloan WBRT Nomogram						**✓**
B12/CRP Index (BCI)	**✓**					**✓**
Bone Metastases Ensemble Trees for Survival (BMETS)						**✓**
Barretos Prognostic Nomogram (BPN)				**✓**		
BPN v2.0				**✓**		
BPN vClin				**✓**		
Biological Prognostic Score (BPS)						**✓**
Chinese Prognostic Index (ChPS)			**✓**			
Chinese Prognostic Prediction Score				**✓**		
Chuang’s Prognostic Scale				**✓**		**✓**
Clinical Prediction Model for 1-Year Mortality				**✓**		
Combined Initial PPI and Δscore (PPI Change Model)						**✓**
Cochin Risk Index Score (CRIS)			**✓**			
Diagnostic Model for Impending Death within 3 Days		**✓**				
Delirium-Palliative Prognostic Score (D-PaP)		**✓**				
Dutch Bone Metastasis Scoring System	**✓**					**✓**
Epic Epic End of Life Care Index (EOLCI)						**✓**
Feliu Prognostic Nomogram (FPN)					**✓**	**✓**
Gastric Cancer Nomogram (Kim et al.)		**✓**				
Gastric Cancer Palliative Chemotherapy Nomogram					**✓**	
Gastrointestinal Cancer Home Hospice Nomogram (non-lab)			**✓**			
Gastrointestinal Malignancy Laboratory Prognostic Score (GI-LPS)			**✓**			
Glasgow Prognostic Score (GPS)						**✓**
Graphic tool for individualised survival curves		**✓**				
Imminent Mortality Predictor in Advanced Cancer (IMPAC)			**✓**			
Japan Palliative Oncology Study – Prognostic Index (JPOS-PI)					**✓**	
Kim Prognostic Model (unnamed)	**✓**					
Kripp et al. model	**✓**					**✓**
Laboratory Bone Metastases (LabBM) Score						**✓**
Laboratory-free nomogram			**✓**			
Laboratory Prognostic Score (LPS)				**✓**		
Lung bone metastases nomogram					**✓**	
Multidisciplinary Team adapted Palliative Prognostic Score (MDT-adapted-PaP)						**✓**
Modified Glasgow Prognostic Score (mGPS)						**✓**
Modified Glasgow Prognostic Score-Performance Status (mGPS-PS)					**✓**	
Modified Objective Prognostic Score (mOPS-A)			**✓**			**✓**
mOPS-B			**✓**			**✓**
National Cancer Centre Singapore (NCCS) Prognostic Model			**✓**			
new-ChPS	**✓**					
Objective Prognostic Index for Advanced Cancer (OPI-AC)		**✓**				
Objective Palliative Prognostic Score (OPPS)	**✓**					
Objective Prognostic Score (OPS)	**✓**					**✓**
OPS (Okugawa variant)				**✓**		
Prediction of 1-day Survival-Survival Decision Tree	**✓**					
P3did-Dcision Tree		**✓**				
P3did Score		**✓**				
Palliative Prognostic Score (PaP)	**✓**					**✓**
PaP Score Nomogram					**✓**	
Prognosis in Palliative Care Study (PiPS-A)		**✓**				**✓**
PiPS-B		**✓**				**✓**
Palliative Prognostic Index (PPI)				**✓**		**✓**
Palliative Performance Scale (PPS)-based 90-Day Mortality Model						**✓**
Palliative Radiotherapy and Inflammation Study (PRAIS) predictive models		**✓**				
Prognostic Risk Model for patients with Advanced Cancer (PRO-MAC)			**✓**			
Prognostic Index for Advanced Pancreatic Cancer		**✓**				
Prognostic model for survival prediction for patients with advanced cancer in a palliative care setting				**✓**		
Prognostic model using routine laboratory results	**✓**					
Prognostic Nomogram for Advanced Cancer in Palliative Care (Zhao nomogram)				**✓**		
Prognostic scale for 7-day survival prediction				**✓**		
PRONOPALL Score						**✓**
Performance Status-based Palliative Prognostic Index (PS-PPI)		**✓**				
Laboratory Prognostic Score for Respiratory Malignancy (R-LPS)			**✓**			
Rades et al. three-tiered survival score	**✓**					**✓**
Recursive Partitioning (RP) Prognostic Model					**✓**	
Risk Scoring System for 6-Month Mortality in Gastric Cancer with Peritoneal Carcinomatosis	**✓**					
Rapid Response Radiotherapy Program (RRRP) 3-variable Number of Risk Factors (NRF)/Survival Prediction Score (SPS) Model					**✓**	**✓**
RRRP 6-variable NRF/SPS Model	**✓**					**✓**
Simple Clinical Score (SCS)	**✓**					
Schonwetter Terminal Lung Cancer Prognostic Model				**✓**		
Simplified Palliative Prognostic Index (sPPI)	**✓**					**✓**
Stone Prognostic Index (unnamed)		**✓**				
Survival score for elderly patients (≥ 65 years) with bone metastases receiving radiotherapy (unnamed)			**✓**			
Type of cancer, ECOG Performance Status, Age, prior palliative (TEACHH) Model	**✓**					**✓**
Tournoux-Facon Hepatocellular Carcinoma Palliative Score					**✓**	

Of the 77 models identified, 8 (10%) had undergone development only, with no evidence of subsequent internal or external validation in the included literature.

Thirty-four (44%) had been developed and internally validated but had not been evaluated in an independent external population within the included literature. Of these, 13 were internally validated using resampling techniques, such as bootstrapping or cross-validation; 10 used a random split-sample approach; and 10 used a non-random split-sample approach.

Thirty-five models (46%) had been externally validated in at least one independent population. Nine models (12%) underwent validation within the same study or as part of a simultaneous development and validation study, with two of these subsequently subjected to further independent external validation. Nine models (12%) had been developed in a study captured within this review and subsequently validated in at least one independent external population in a separate study. Six models (8%) had a development and internal validation study captured in this review and had also been externally validated in at least one independent population. The remaining 11 models (14%) appeared only in external validation studies within this review, meaning their original development studies were not captured, suggesting their development literature falls outside the scope of this review’s inclusion criteria (e.g., developed for a different population to which it was then externally validated).

External validation activity was heavily concentrated on a small subset of models. The Palliative Prognostic Index (PPI) was the most extensively validated model, with 16 external validation studies across 10 countries [106, 108–121, 132], followed by the Rapid Response Radiotherapy Program (RRRP) 3-variable Number of Risk Factors (NRF)/Survival Prediction Score (SPS) (6 external validation studies across 4 countries), the Palliative Prognostic Score (PaP) (5 external validation studies across 4 countries), and the Objective Prognostic Score (OPS) (4 external validation studies across 3 countries). Twenty-two of the 35 externally validated models (63%) had been evaluated only once, and only within a single country.

A summary of externally validated models, including the countries, settings, and populations in which they were validated, is provided in Additional File 4.

## Discussion

### Summary of evidence

This scoping review provides the most comprehensive mapping to date of end-of-life prognostic models developed, validated, or evaluated for impact in adults with advanced cancer. A recent systematic review and meta-analysis by Fung et al. [15] pooled discriminative performance data for externally validated models in advanced solid tumour patients, providing valuable quantitative estimates for the most frequently studied tools.

However, that review did not capture the full landscape of model development, internal validation, or impact evaluation activity in this field, nor did it include models evaluated in haematological malignancies or single-cancer cohorts. The present scoping review addresses these gaps by mapping the breadth of prognostic modelling activity across all phases of the prognostic model development framework, offering a complementary evidence synthesis to inform both research priorities and clinical decision-making. Across 106 studies, 77 distinct models were identified, spanning a wide range of populations, settings, predictors, and time horizons.

Three key findings emerged from this review. First, the evidence base is relatively well developed in terms of validation: half of included studies (50%) contained an external validation component, and nearly half of identified prognostic models (46%) had undergone external validation. This contrasts with reviews of prediction model research in other healthcare fields, in which development and/or internal validation studies have generally been more common than external validation studies [133, 134].

Second, although external validation studies were common, validation efforts were concentrated on a small number of models; 63% of the 77 identified models had only been evaluated in the settings in which they were developed, limiting confidence in their wider generalisability [135]. This concentration is consistent with the findings of Fung et al. [15], whose meta-analysis identified PPI, PaP, and OPS as the most extensively studied tools, mirroring our own results, in which PPI, PaP, and OPS were some of the most frequently externally validated models. While 44% of models had undergone internal validation, this provides only limited reassurance, as internal validation helps assess overfitting but cannot determine whether a model will perform adequately in new populations or settings [136].

This could explain why, despite the availability of multiple prediction models, clinicians have not been adopting them routinely in clinical practice and many are still relying on their clinical judgement alone [6, 16, 137].

Third, and most importantly, no studies evaluated the impact of prognostic model use on clinical decision-making or patient outcomes. This absence is consistent with wider prognostic modelling literature [7, 138–140]. This is particularly important, as impact studies provide the strongest evidence that a model improves care [7, 13]. Good statistical performance alone is insufficient: a model may accurately distinguish between shorter- and longer-term survival without changing communication, referral patterns, or treatment decisions. The lack of impact evidence therefore represents not simply a gap in the literature, but a major barrier to the clinical implementation of prognostic models in advanced cancer. This conclusion is further reinforced by Fung et al. [15], who found that all externally validated models in their meta-analysis carried a high risk of bias and concluded that further validation and implementation studies are needed before the clinical utility of even the best-performing models can be confirmed. Taken together, the quantitative synthesis of Fung et al. [15] and the broader landscape mapped by this scoping review converge on the same conclusion: statistical performance data, while necessary, are insufficient to justify routine clinical implementation, and impact evaluation must now be treated as the field’s top research priority.

Several factors may explain this absence of impact studies in advanced cancer. Impact evaluation is methodologically demanding and costly, as demonstrating true clinical benefit usually requires large, prospective, and often randomised studies [141]. Conducting such research in end-of-life populations also raises ethical and practical challenges, including concerns about participant burden, consent, and the sensitivity of studying prognostic interventions in people with limited life expectancy [142]. In addition, there is no clear consensus on how the impact of prognostic models should be assessed, including which outcomes are most meaningful to patients and their families [23]. Addressing these methodological, ethical, and conceptual challenges should be a priority for future research if this important evidence gap is to be closed.

This review also highlights important gaps in population and geographic coverage. Studies from LMICs were markedly underrepresented, with only eleven studies conducted in Brazil, Kuwait, Egypt, Saudi Arabia, Indonesia, India, and Thailand. Given that LMICs account for approximately 65% of cancer-related deaths worldwide [143], this is both a research gap and a health equity issue. Models developed in high-income countries may rely on predictors that are not routinely available in resource-limited settings. In addition, most studies included mixed-cancer cohorts (80%), leaving limited evidence for tumour-specific models, particularly in haematological malignancies, where prognostication presents distinct challenges due to the fluctuating illness trajectory [144].

Considerable heterogeneity was also evident in prognostic time horizons, which ranged from next-day survival to 12-month mortality. Some models focused on imminent death, while others estimated survival at 30 days, 3 months, or 6–12 months. This variation likely reflects the different clinical decisions that prognostic models are intended to support, from immediate care planning to longer-term service allocation, as well as inconsistencies in definitions for “end-of-life” [145].

### Implications for research and practice

These findings have several implications. As prognostic methods continue to evolve rapidly with the rise of Artificial Intelligence, and new prognostic models are developed, the field should prioritise independent external validation of developed models across diverse settings, healthcare systems, and underrepresented populations, particularly LMICs and disease-specific cohorts. Without external validation, it remains uncertain whether these models perform reliably outside the populations in which they were derived, limiting confidence in their generalisability and suitability for wider clinical use [135]. Furthermore, with 77 models already identified, many developed for similar populations and outcomes, researchers and funders should also consider the risk of unnecessary duplication; before developing new models, systematic consideration of whether existing tools have been adequately developed and validated in the target population is warranted.

Importantly, the volume of external validation studies associated with a given model should not in itself be taken as evidence of methodological robustness. Formal methodological quality appraisal was outside the scope of this scoping review; therefore future systematic reviews in this field should incorporate formal methodological quality assessment using the Prediction model Risk Of Bias ASsessment Tool (PROBAST) to evaluate the risk of bias and applicability of both development and validation studies, and to identify which models are sufficiently well-developed to warrant impact evaluation [146]. Reporting quality of included studies should also be assessed against contemporary reporting guidelines; the recently published TRIPOD-AI extension to the TRIPOD statement provides updated guidance for the transparent reporting of prediction models developed using machine learning and artificial intelligence methods [147], and its application in future systematic reviews would further improve methodological transparency in this field.

In addition, impact evaluation should now be treated as a research priority. Prognostic models should not be implemented in routine care on the basis of statistical performance alone. Evidence from other areas of healthcare shows that models may perform well in validation studies yet fail to improve care, or even cause harm, when introduced into practice [148]. Implementation should therefore be preceded by impact evaluation to determine whether model use meaningfully influences clinical decision-making, care processes, or patient outcomes. Funding bodies, research networks, and professional societies should support the development of consensus on appropriate study designs, relevant outcomes, and ethical frameworks for evaluating prognostic models in advanced cancer.

The field would also benefit from methodological guidance on feasible approaches to impact evaluation in this setting, including which outcomes should be measured and how. Typically, cluster randomised trials are considered the scientifically strongest way to assess the impact of prognostic models [7]. Examples from other clinical field include the STarTBack trial [149] and the Pneumonia Severity Index [150]. However, pragmatic designs can also generate meaningful evidence of clinical benefit, as demonstrated by the before-and-after evaluation of the Ottawa Ankle Rules [151]. This suggests that pragmatic, lower-resource designs may offer a viable starting point in end-of-life populations where recruitment and randomisation present additional ethical and logistical challenges.

For clinicians already using prognostic models in advanced cancer care, this review provides a useful overview of the tools currently available and the evidence supporting them. However, the sheer number of models identified may itself present a barrier to adoption; faced with such choice, clinicians may find it difficult to identify which tool is most appropriate for their patient population and setting. Among existing models, the PPI, PaP, and OPS appear to have the strongest external validation evidence and may therefore represent the most defensible options for clinical use pending impact evaluation. However, in the absence of such evidence, these models should be applied cautiously and used to support, rather than replace, CPS.

### Strengths and limitations

To the authors’ knowledge, this is the first scoping review to comprehensively map prognostic model research in advanced cancer across the prediction model development framework described by PROGRESS [7–10]. The review was guided by a pre-registered protocol and informed by a comprehensive search of four databases, supplemented by citation searching and grey literature sources. The breadth of evidence synthesised, spanning three decades of research, further strengthens the comprehensiveness of this review. Several limitations should, however, be acknowledged. Restricting the review to English-language publications may have led to the exclusion of relevant studies, particularly from countries such as Japan and other East Asian settings where prognostic modelling research appears to be relatively common, as well as from low- and middle-income countries, which were underrepresented in the included literature. In keeping with scoping review methodology, formal critical appraisal of included studies was not undertaken; consequently, no conclusions can be drawn regarding methodological quality, risk of bias, or certainty of evidence. Finally, given the rapidly evolving nature of prognostic model research, some recently developed or published models may also have been missed, particularly those emerging after the final search update.

## Conclusions

This scoping review mapped the scope and characteristics of prognostic model research in adults with advanced cancer, identifying 77 distinct models across 106 studies conducted in 31 countries over three decades. Although the evidence base includes a substantial number of external validation studies, these have been concentrated on a relatively small subset of models. The most important finding is the complete absence of impact studies, representing a major barrier to the responsible clinical implementation of prognostic models in advanced cancer care. Future research should prioritise independent external validation of existing models across diverse settings and populations, particularly in LMICs and underrepresented disease-specific cohorts. Most importantly, the field now requires well-designed impact evaluations to determine whether use of these models improves clinical decision-making, care processes, or outcomes for patients approaching the end-of-life. Until such evidence is available, routine clinical adoption should remain cautious, with prognostic models used to support rather than replace clinicians’ prognostic assessment.

## Supplementary Information

Supplementary Material 1.

Supplementary Material 2.

Supplementary Material 3.

Supplementary Material 4.

## Data Availability

The dataset(s) supporting the conclusions of this article is(are) included within the article (and its additional file(s)).
