# Supplementary material for: End-of-life prognostic models in advanced cancer: a scoping review of model development, validation, and impact
Source: BMC Palliat Care. 2026 Jul 3;25:280. doi: 10.1186/s12904-026-02172-3 (PMC13599129; doi:10.1186/s12904-026-02172-3)
Supplement: Supplementary file 1 [file 12904_2026_2172_MOESM1_ESM.docx]

| **#** | **Concept** | **Search terms** |
| --- | --- | --- |
| 1 | Advanced cancer | exp Neoplasms/ or (neoplas* or cancer* or carcinoma* or mass* or malignan* or oncolog* or carcinoma* or sarcoma* or tumor* or tumour* or lesion* or metasta* or nodule*).tw. |
| 2 |  | exp Palliative Care/ or exp Terminal Care/ or hospices/ or (advanced adj2 disease*).tw. or (advanced adj2 illness*).tw. or (terminal* adj2 care).tw. or (palliat* or terminal* or metasta* end stage or late stage or incurable or life limiting or end of life or stage IV or unresectable or terminal* ill* or hospice care).tw. |
| 3 | End-of-life predictions | (exp Prognosis/ and (exp mortality/ or exp survival/ or exp survival analysis/)) or ((predict* or prognos*) adj3 (survival or mortality or end of life or life expectancy or "advanced cancer")).tw. |
| 4 | Prognostic models | (scor* or tool* or scale* or model* or instrument* or assess* or index or indices or scale* or algorithm* or tests* or nomogram*).tw. |
| 5 | Statistical terms | (((logistic or statistic*) adj3 model*) or (c-index or concordance index or c-statistic or concordance statistic or Area under the curve or AUC or calibrat* or discrimat* or validat* or impact or (develop* adj3 model*) or (calibrat* adj3 model*) or (discriminat* adj3 model*) or (validat* adj3 model*) or ((Validat* and (cohort* or set* or internal or external)) or ((impact or effect* or outcome* or benefit* or burden* or issue*) adj3 model*) or (impact and (assessment or trial or study or evaluation))))).tw. |
| 6 | Boolean operators | 1 AND 2 AND 3 AND 4 AND 5 |
| 7 | Limits | 6 not (conference abstract or conference paper or conference review or letter or editorial or note or comment or meta analysis or review or systematic review or scoping review).pt. |
| 8 | Limits | limit 7 to (english language and ("young adult (19 to 24 years)" or "adult (19 to 44 years)" or "young adult and adult (19-24 and 19-44)" or "middle age (45 to 64 years)" or "middle aged (45 plus years)" or "all aged (65 and over)" or "aged (80 and over)")) |

**Additional File 1.** Search strategy for the scoping review.

This strategy was employed on OVID MEDLINE and modified for other databases.
