# Supplementary material for: End-of-life prognostic models in advanced cancer: a scoping review of model development, validation, and impact
Source: BMC Palliat Care. 2026 Jul 3;25:280. doi: 10.1186/s12904-026-02172-3 (PMC13599129; doi:10.1186/s12904-026-02172-3)
Supplement: Supplementary file 2 [file 12904_2026_2172_MOESM2_ESM.docx]

**Additional File 2.** Characteristics of included studies (n = 106).

| **Authors** | **Country of study** | **Cohort type** | **Model name/**  **description** | **Predictive model development framework** | **Sample size** | **Clinical setting/s (n)** | **Setting type** | **Treatment status** | **Cancer site** |
| --- | --- | --- | --- | --- | --- | --- | --- | --- | --- |
| Adelson et al. 2018 (56) | USA | Retrospective cohort study | IMPAC | D + IV *(random split sample)* | D = 468 IV = 201 | Cancer centre (n = 1) | Inpatient | NOT REPORTED | Mixed |
| Alshemmari et al. 2012 (109) | Kuwait | Prospective cohort study | PPI | EV | 91 | Cancer hospital (n = 1) | Inpatient | No ongoing anticancer treatment (palliative or supportive care only) | Mixed |
| Alsirafy et al. 2016 (92) | Egypt | Prospective cohort study | Chuang’s prognostic scale | EV | 36 | Oncology clinic/cancer centre (n = 1) | Outpatient | Palliative systemic anticancer therapy | Metastatic colorectal cancer |
| Al-Zahrani et al. 2012 (93) | Saudi Arabia | Prospective cohort study | Chuang’s prognostic scale | EV | 61 | Oncology ward in a hospital (n = 1) | Inpatient | No ongoing anticancer treatment (palliative or supportive care only) | Mixed |
| Arkin et al. 2020 (45) | Turkey | Retrospective cohort study | ANN model for 30-day survival | D + IV *(resampling)* | 189 | PCU (n = 1) | Inpatient | No ongoing anticancer treatment (palliative or supportive care only) | Mixed |
| Barbot et al. 2008 (28) | France | Prospective cohort study | 2-Month Prognostic Score (Barbot score) | D | 177 | Hospitals (n =2) | Inpatient | NOT REPORTED | Mixed |
| Bourgeois et al. 2017 (123) | France | Prospective multicentre observational study | PRONOPALL score | EV | 262 | Oncology centres (n = 16) | Outpatient | Palliative anticancer therapy (chemotherapy, TKI, or monoclonal antibodies) | Mixed |
| Chen et al. 2015 (29) | Taiwan | Retrospective cross-sectional study | OPPS | D | 234 | Hospice ward in a hospital (n = 1) | Inpatient | NOT REPORTED | Mixed |
| Chen et al. 2018 (120) | Brazil | Prospective observational study | PPI;  RRRP 3-variable NRF/SPS Model | EV | 333 | Cancer centre (n = 1) | Inpatient | Receiving palliative radiotherapy | Mixed |
| Cheng et al. 2012 (110) | Taiwan | Retrospective cohort study | PPI | EV | 623 | Hospice consultation service in a medical centre (n = 1) | Inpatient | No ongoing anticancer treatment (palliative or supportive care only) | Mixed |
| Chiang et al. 2009 (67) | Taiwan | Prospective cohort study | Prognostic scale for 7-day survival prediction | D + IV  *(non-random split sampling)* | D = 374  IV = 353 | Hospice ward in a hospital (n = 1) | Inpatient | No ongoing anticancer treatment (palliative or supportive care only) | Mixed |
| Chiang et al. 2017 (46) | Taiwan | Prospective cohort study | Graphic tool to estimate individualised survival curves for advanced cancer patients in hospice care | D + IV *(resampling)* | 286 | Hospice ward in a hospital (n = 1) | Inpatient | No ongoing anticancer treatment (palliative or supportive care only) | Mixed |
| Chow et al. 2002 (30) | Canada | Prospective cohort study | RRRP 6-variable NRF/SPS Model | D | 395 | Palliative radiotherapy clinic in a cancer centre (n = 1) | Outpatient | Palliative radiotherapy | Mixed |
| Chow et al. 2006 (95) | Canada | Prospective cohort study | Dutch Bone Metastasis Scoring System;    RRRP 6-variable NRF/SPS Model | EV | 231 | Palliative radiotherapy clinic in a cancer centre (n = 1) | Outpatient | Palliative radiotherapy | Mixed (spinal bone metastases) |
| Chow et al. 2008 (80) | Canada | Prospective cohort study | RRRP 3-variable NRF/SPS Model | D + EV  *(in same study)* | D = 395 TV = 445 EV = 467 | Palliative radiotherapy clinic in a cancer centre (n = 2) | Outpatient | Palliative radiotherapy | Mixed |
| Chow et al. 2009a (128) | Canada | Prospective cohort study | RRRP 6-variable NRF/SPS Model | EV | 445 | Palliative radiotherapy clinic in a cancer centre (n = 1) | Outpatient | Palliative radiotherapy | Mixed |
| Chow et al. 2009b (81) | Canada | Prospective cohort study | Recursive Partitioning (RP) Prognostic Model | D + EV  *(in same study)* | D = 395 TV = 445 EV = 468 | Palliative radiotherapy clinic in a cancer centre (n = 2) | Outpatient | Palliative radiotherapy | Mixed |
| Chow et al. 2011 (125) | USA Canada | Secondary analysis of RCT | RRRP 3-variable NRF/SPS Model | EV | 908 | Palliative radiation facilities (n = 66) | Outpatient | Palliative radiotherapy | Breast or prostate with bone metastases |
| Chuang et al. 2004 (68) | Taiwan | Prospective cohort study | Chuang’s Prognostic Scale | D + IV | D = 356 IV = 184 | PCU (n = 1) | Inpatient | NOT REPORTED | Mixed |
| Cui et al. 2014 (31) | China | Prospective cohort study | New Chinese Prognostic Scale (new-ChPS) | D | 320 | Hospitals (n = 12) | Inpatient | No ongoing anticancer treatment (palliative or supportive care only) | Mixed |
| Dosani et al. 2018 (130) | Canada | Retrospective cohort study | TEACHH model | EV | 180 | Cancer centre (n=1) | Outpatient | Palliative radiotherapy | Mixed with spinal metastases |
| Durand et al. 2012 (57) | France | Prospective cohort study | CRIS | D + IV *(random split sample)* | D = 334 IV = 166 | PCU (n = 1) | Inpatient | No ongoing anticancer treatment (palliative or supportive care only) | Mixed |
| Elledge et al. 2021 (91) | USA | Retrospective cohort study | BMETS | EV | 182 | Radiation oncology clinic in a hospital (n = 2) | Outpatient | Palliative radiotherapy | Mixed with bone metastases |
| Ermacora et al. 2019 (106) | Italy | Prospective cohort study | PaP;  OPS;  PPI | EV | 334 | PCUs (n = 2) | Inpatient | No ongoing anticancer treatment (palliative or supportive care only) | Mixed |
| Feliu et al. 2011 (82) | Spain | Prospective cohort study | FPN | D + EV  *(in same study)* | D = 406  EV = 474 | Oncology and palliative care units (n = 11) | Inpatient and outpatient | No ongoing anticancer treatment (palliative or supportive care only) | Mixed |
| Geissbühler et al 2000 (32) | Switzerland | Prospective cohort study | BCI | D | 161 | PCU (n = 1) | Inpatient | NOT REPORTED | Mixed |
| Gensheimer et al. 2025 (96) | USA | Retrospective cohort study | Epic EOLCI | EV | 1399 | Oncology outpatient clinic in a hospital (n = 4) | Outpatient | NOT REPORTED | Mixed |
| Glare et al. 2014 (126) | USA | Retrospective cohort study | RRRP 3-variable NRF/SPS Model | EV | 237 | Pain and palliative care outpatient clinic in a cancer centre (n = 1) | Outpatient | No ongoing anticancer treatment (palliative or supportive care only) | Mixed |
| Gwilliam et al. 2011 (47) | UK | Prospective cohort study | PiPs-A;  PiPS-B | D + IV *(resampling)* | 1018 | Palliative care services (n = 18) | Inpatient, outpatient, and community/home-based | No ongoing anticancer treatment (palliative or supportive care only) | Mixed |
| Hamano et al. 2015 (33) | Japan | Prospective cohort study | sPPI | D | 2363 | PCUs (n = 16)  Hospital-based palliative care teams (n = 19)  Home-based palliative care services (n = 23) | Inpatient, outpatient, and community/home-based | Palliative chemotherapy, hormone therapy, and/or radiotherapy | Mixed |
| Hamano et al. 2018 (48) | Japan | Secondary analysis of prospective cohort study | OPI-AC | D + IV *(resampling)* | 1039 | Palliative care teams (n = 19)  PCUs (n = 16)  Home-based palliative care (HPC) (n = 23) | Inpatient, outpatient, and community/home-based | Palliative chemotherapy and/or radiotherapy | Mixed |
| Hiratsuka et al. 2024 (102) | Taiwan | Secondary analysis of prospective cohort study | mOPS-A;  mOPS-B | EV | 317 | PCUs (n = 4) | Inpatient | No ongoing anticancer treatment (palliative or supportive care only) | Mixed |
| Huang et al. 2014 (69) | China | Prospective cohort study | Chinese Prognostic Prediction Score (no formal name given) | D + IV | D = 181 IV = 128 | Hospitals (n = 2) | Inpatient | No ongoing anticancer treatment (palliative or supportive care only) | Mixed |
| Hui et al. 2015 (78) | USA  Brazil | Prospective cohort study | Diagnostic Model for Impending Death within 3 Days | D + IV  *(resampling)* | 322 | Acute PCUs (n = 356) | Inpatient | NOT REPORTED | Mixed |
| Hum et al. 2020 (58) | Singapore | Prospective cohort study | PRO-MAC | D + IV *(random split sample)* | 840 | PCU (n = 1) | Inpatient | Palliative chemotherapy and/or radiotherapy | Mixed |
| Hyodo et al. 2010 (83) | Japan | Prospective cohort study | JPOS-PI | D + EV  *(in same study)* | D = 201  EV = 208 | PCU (n = 14) | Inpatient | No ongoing anticancer treatment (palliative or supportive care only) | Mixed |
| Iizuka-Honma et al. 2022 (111) | Japan | Retrospective cohort study | PPI | EV | 143 | Haematology ward in a hospital (n = 1) | Inpatient | Palliative chemotherapy | Haematologic malignancies |
| Inomata et al. 2014 (112) | Japan | Retrospective cohort study | PPI | EV | 165 | Hospital (n = 1) | Inpatient | No ongoing anticancer treatment (palliative or supportive care only) | Lung |
| Jho et al. 2016 (103) | Japan | Prospective cohort study | OPS | EV | 1146 | Palliative care teams (n = 19)  PCUs (n = 16)  Home-based palliative care (HPC) (n = 23) | Inpatient, outpatient, and community/home-based | No ongoing anticancer treatment (palliative or supportive care only) | Mixed |
| Kadokura et al. 2020 (113) | Japan | Retrospective cohort study | PPI;  BPS | EV | 354 | Hospital (n = 1) | Inpatient | Non-curative (palliative) | GI |
| Kao et al. 2014 (94) | Taiwan | Prospective cohort study | Combined Initial PPI and Δscore (PPI Change Model) | EV | 2392 | Palliative care consultation service in a hospital (n = 1) | Inpatient | No ongoing anticancer treatment (palliative or supportive care only) | Mixed |
| Kawai et al. 2018 (70) | Japan | Retrospective cohort study | LPS | D + IV  *(non-random split sampling)* | D = 761 IV = 547 | Hospice (n = 1) | Inpatient | No ongoing anticancer treatment (palliative or supportive care only) | Mixed |
| Kelly et al. 2007 (89) | UK | Prospective cohort study | BCI | EV | 329 | Hospitals (n = 3)  Hospices (n = 4) | Inpatient and outpatient | No ongoing anticancer treatment (palliative or supportive care only) | Mixed |
| Kikuchi et al. 2007 (71) | Japan | Retrospective cohort study | Prognostic model for survival prediction for patients with advanced cancer in a palliative care setting | D + IV  *(non-random split sampling)* | D = 238  IV = 90 | PCU (n = 1) | Inpatient | NOT REPORTED | Mixed |
| Kim et al. 2014 (121) | South Korea | Retrospective cohort study | PPI;  PaP | EV | 415 | Hospices (n = 2) | Inpatient | No ongoing anticancer treatment (palliative or supportive care only) | Mixed |
| Kim et al. 2018 (49) | South Korea | Retrospective cohort study | Gastric Cancer Nomogram | D + IV *(resampling)* | 949 | Tertiary cancer centre (n = 1) | Outpatient | Palliative chemotherapy | Gastric |
| Kim et al. 2022 (34) | South Korea | Prospective cohort study | Kim Prognostic Model (unnamed) | D | 200 | Oncology clinic in a cancer centre (n = 1) | Outpatient | Palliative chemotherapy | Mixed |
| Kou et al. 2016 (50) | Japan | Retrospective cohort study | Prognostic Index for Advanced Pancreatic Cancer | D + IV *(resampling)* | 306 | Hospitals (n = 2) | Outpatient | Palliative chemotherapy | Pancreatic |
| Kripp et al. 2014 (35) | Germany | Retrospective analysis of prospective cohort study | Kripp et al. model | D | 290 | PCUs (n = 1) | Inpatient | NOT REPORTED | Haematologic malignancies |
| Krishnan et al. 2013 (36) | USA | Retrospective cohort study | TEACHH model | D | 862 | Oncology radiation clinics (n = 2) | Outpatient | Palliative radiotherapy | Mixed |
| Laird et al. 2013 (84) | Denmark Finland Germany UK Iceland Italy Lithuania Norway Sweden Switzerland Greece | Prospective cohort study | mGPS-PS | D + EV  *(in same study)* | D = 1825 EV = 631 | Oncology wards (n = NOT REPORTED)  PCUs/hospices (n = NOT REPORTED)  Surgical wards (n = NOT REPORTED)  Outpatient departments (n = NOT REPORTED) | Inpatient and outpatient | Palliative chemotherapy | Mixed |
| Li et al. 2025 (59) | China | Retrospective cohort study | Laboratory-free nomogram | D + IV *(random split sample)* | D = 3163 IV = 1066 | Hospice unit (n = 1) | Inpatient | No ongoing anticancer treatment (palliative or supportive care only) | Mixed |
| Lingjun et al. 2009 (60) | China | Retrospective cohort study | ChPS | D + IV *(random split sample)* | D = 814  IV = 205 | Palliative care home services (n = 1) | Home-based | NOT REPORTED | Mixed |
| Lojanapiwat et al. 2021 (100) | Australia | Retrospective cohort study | mGPS | EV | 170 | Hospital (n = 1) | Inpatient | NOT REPORTED | Mixed |
| Maltoni et al. 1999 (107) | Italy | Prospective cohort study | PaP | EV | 451 | Palliative care centres (n = 14) | Inpatient, outpatient, and home-based | Palliative steroid treatment and/or hormone therapy | Mixed |
| Mei et al. 2013 (122) | Singapore | Prospective cohort study | PPS-based 90-Day Mortality Model | EV | 296 | PCUs (n = 1) | Inpatient | NOT REPORTED | Mixed |
| Mendis et al. 2015 (99) | Australia | Retrospective cohort study | MDT-adapted PaP | EV | 644 | Palliative care consultation service in a cancer centre (n = 1) | Inpatient and outpatient | NOT REPORTED | Mixed |
| Miura et al. 2015 (97) | Japan | Prospective cohort study | GPS | EV | 1160 | PCUs (n = 16)  Hospital-based palliative care teams (n = 19)  Home-based palliative care services (n = 23) | Inpatient and home-based | No ongoing anticancer treatment (palliative or supportive care only) | Mixed |
| Miyagi et al. 2021 (37) | Japan | Retrospective cohort study | Prognostic model using routine laboratory results | D | 225 | PCU (n = 1) | Inpatient | No ongoing anticancer treatment (palliative or supportive care only) | Mixed |
| Mojica-Marquez et al. 2020 (131) | USA | Retrospective cohort study | TEACHH model;  RRRP 3-variable NRF/SPS Model | EV | 505 | Integrated cancer network (academic and community practices) | Inpatient and outpatient | Palliative radiotherapy | Mixed |
| Mori et al. 2021 (79) | Japan | Prospective cohort study | P3did-Decision Tree  P3did Score | D + IV  *(resampling)* | 1396 | PCUs (n = 23) | Inpatient | NOT REPORTED | Mixed |
| Mori et al. 2025 (38) | Japan | Prospective cohort study | P1d-Survival Decision Tree | D | 1396 | PCUs (n = 23) | Inpatient | NOT REPORTED | Mixed |
| Morita et al. 1999 (72) | Japan | Retrospective cohort study | PPI | D + IV  *(non-random split sampling)* | D = 150  IV = 95 | PCU (n = 1) | Inpatient | NOT REPORTED | Mixed |
| Nabal et al. 2014 (51) | Spain | Prospective analytical study | 7- and 30-Day Survival CART Model | D + IV *(resampling)* | 698 | Palliative care home teams (n = NOT REPORTED) | Community/home-based | NOT REPORTED | Mixed |
| Nagai et al. 2022 (61) | Japan | Retrospective cohort study | GI-LPS | D + IV *(random split sample)* | D = 490 IV = 326 | Hospice (n = 1) | Inpatient | No ongoing anticancer treatment (palliative or supportive care only) | GI |
| Nieder et al. 2017a (90) | Norway Germany | Retrospective cohort study | Barnholtz-Sloan WBRT Nomogram | EV | 254 | Radiotherapy department in a hospital (n = 2) | Inpatient and outpatient | Palliative radiotherapy | Mixed with brain metastases |
| Nieder et al. 2017b (124) | Norway | Retrospective cohort study cohort study | Rades et al. three-tiered survival score | EV | 232 | Radiotherapy department in a hospital (n = 1) | Inpatient and outpatient | Palliative radiotherapy | Lung |
| Nieder et al. 2021 (98) | Norway | Retrospective cohort study | LabBM score | EV | 375 | Radiotherapy department in a hospital (n = 1) | Inpatient and outpatient | Palliative radiotherapy | Mixed |
| Ohno et al. 2017 (114) | Japan | Retrospective | PPI;  Kripp et al. model | EV | 114 | Haematology ward in a hospital (n = 1) | Inpatient | Palliative chemotherapy and/or radiotherapy | Haematological |
| Okugawa et al. 2017 (73) | Japan | Retrospective cohort study | OPS (Okugawa variant) | D + IV  *(non-random split sampling)* | D = 97 IV = 90 | PCU (n = 1) | Inpatient | No ongoing anticancer treatment (palliative or supportive care only) | Mixed |
| Owusuaa et al. 2022 (151) | The Netherlands | Prospective cohort study | Clinical Prediction Model for 1-Year Mortality (3 versions: Simple, Clinical, Extended) | D + IV  *(non-random split sampling)* | 867 | Hospitals (n = 6) | Inpatient and outpatient | Palliative care/treatment (not specified) | Mixed |
| Paiva et al. 2018 (74) | Brazil | Prospective cohort study | BPN | D + IV  *(non-random split sampling)* | D = 221 IV = 276 | Palliative care outpatient clinic (n = 1) | Outpatient | Palliative anti-neoplastic treatments (not specified) | Mixed |
| Partridge et al. (2012) (101) | UK | Retrospective cohort study | mGPS | EV | 102 | Hospice (n = 1) | Inpatient | NOT REPORTED | Mixed |
| Pereira et al. 2024 (39) | Brazil | Retrospective cohort study | Risk Scoring System for 6-Month Mortality in Gastric Cancer with Peritoneal Carcinomatosis | D | 211 | Hospital (n=1) | Inpatient | Palliative surgery and chemotherapy | Gastric cancer with peritoneal carcinomatosis |
| Pirovano et al. 1999 (40) | Italy | Prospective cohort study | PaP | D | 519 | Palliative care/ oncology centres (n = 22) | Inpatient and outpatient | Palliative steroid treatment and/or hormone therapy | Mixed |
| Preto et al. 2022 (75) | Brazil | Secondary analysis of two prospective cohort studies | BPN v2.0;  BPN vClin | D + IV  *(non-random split sampling)* | D = 215 IV = 276 | Palliative care outpatient clinic (n = 1) | Outpatient | Palliative anti-neoplastic treatments (not specified) | Mixed |
| Rades et al. 2016 (41) | Germany | Retrospective cohort study | Rades et al. three-tiered survival score | D | 125 | Radiation oncology department (n = NOT REPORTED) | NOT REPORTED | Palliative radiotherapy | Lung |
| Rades et al. 2022 (62) | Germany, Denmark, USA | Retrospective cohort study | Survival score for elderly patients (≥65 years) with bone metastases receiving radiotherapy (unnamed) | D + IV *(random split sample)* | D = 174 IV = 174 | Radiation oncology department (n = NOT REPORTED) | NOT REPORTED | Palliative radiotherapy | Mixed with bone metastases |
| Rossi et al. 2024 (52) | Norway, Italy, Spain, UK | Secondary analysis of prospective cohort study | PRAIS predictive models | D + IV *(resampling)* | 567 | Palliative radiotherapy centres (n = 6) | Outpatient | Palliative radiotherapy | Mixed with bone metastases |
| Sakurai et al. 2024 (127) | Japan | Secondary analysis of retrospective and prospective cohort studies | RRRP 3-variable NRF/SPS Model | EV | 787 | Radiation oncology departments (n = 4) | NOT REPORTED | Palliative radiotherapy | Mixed with bone and non-bone-metastasis |
| Scarpi et al. 2011 (53) | Italy | Retrospective cohort study | D-PaP | D + IV *(resampling)* | 361 | Palliative care centres (n = 10) | Inpatient | Palliative hormone therapy and/or radiotherapy | Mixed |
| Scarpi et al. 2022 (85) | Italy | Retrospective cohort study | PaP score nomogram | D + EV  *(in same study)* | D = 519 IV = 451 EV = 549 | Hospices (n = NOT REPORTED) | Inpatient | Palliative hormone therapy and/or radiotherapy | Mixed |
| Schonwetter et al. 1994 (76) | USA | Longitudinal cohort study | Schonwetter Terminal Lung Cancer Prognostic Model | D + IV  *(non-random split sampling)* | D = 310 IV = 78 | Hospice (n = 1) | Community/home-based | No ongoing anticancer treatment (palliative or supportive care only) | Lung |
| Shatri et al. 2021 (115) | Indonesia | Retrospective cohort study | PPI | EV | 160 | Hospital (n = 1) | Inpatient | NOT REPORTED | Mixed |
| Sin et al. 2021 (63) | Singapore | Prospective cohort study | NCCS Prognostic Model | D + IV *(random split sample)* | D = 175  IV = 113 | Radiation oncology department (n = 1) | Inpatient and outpatient | Palliative radiotherapy | Mixed |
| Sonoda et al. 2014 (116) | Japan | Retrospective cohort study | PPI;  PaP | EV | PPI = 247 PaPS = 187 | Palliative care consultation services in a hospital (n = 1) | Inpatient | No ongoing anticancer treatment (palliative or supportive care only) | Mixed |
| Sripongpun et al. 2018 (42) | Thailand | Retrospective cohort study | SCS | D | 98 | Gastroenterology/hepatology unit in a hospital (n = 1) | Inpatient | NOT REPORTED | Malignant distal biliary obstruction |
| Stone et al. 2008a (132) | Republic of Ireland | Prospective cohort study | PPI | EV | 194 | Specialist palliative care service in a hospital (n = 1) | Inpatient, outpatient, and community/home based | Palliative chemotherapy and/or radiotherapy | Mixed |
| Stone et al. 2008b (54) | UK | Prospective cohort study | Stone Prognostic Index (unnamed) | D + IV *(resampling)* | 329 | Hospitals (n = 3)  Hospices (n = 4) | Inpatient, outpatient, and community/home-based | No ongoing anticancer treatment (palliative or supportive care only) | Mixed |
| Stone et al. 2021 (108) | UK | Prospective cohort study | PiPS-A; PiPS-B; PaP; FPN; PPI | EV | 1833 | Palliative care services (n = 27) | Inpatient, outpatient, and community/home-based | No ongoing anticancer treatment (palliative or supportive care only) | Mixed |
| Subramaniam et al. 2013 (117) | UK | Prospective cohort study | PPI | EV | 262 | Hospices (n = 3) | Inpatient | NOT REPORTED | Mixed |
| Subramaniam et al. 2019 (118) | UK | Prospective cohort study | PPI | EV | 1164 | Hospices (n = 10) | Inpatient | NOT REPORTED | Mixed |
| Suh et al. 2010 (43) | South Korea | Prospective cohort study | OPS | D | 209 | Hospitals (n = 6) | Inpatient | No ongoing anticancer treatment (palliative or supportive care only) | Mixed |
| Tanaka et al. 2022 (64) | Japan | Retrospective cohort study | R-LPS | D + IV *(random split sample)* | D = 390 IV = 259 | PCUs (n = 1) | Inpatient | No ongoing anticancer treatment (palliative or supportive care only) | Respiratory |
| Tiwari et al. 2024 (119) | India | Prospective cohort study | PPI | EV | 227 | Palliative care clinic (n = 1) | Outpatient | Palliative chemotherapy and/or radiotherapy | Mixed |
| Tournoux-Facon et al. 2011 (86) | France | Retrospective cohort study | Tournoux-Facon HCC Palliative Score | D + EV  *(in same study)* | D = 416  EV = 271 | Palliative care settings (n = NOT REPORTED) | Inpatient, outpatient, and community/home-based | No ongoing anticancer treatment (palliative or supportive care only) | Hepatocellular carcinoma |
| van der Linden et al. 2005 (44) | The Netherlands | Secondary analysis of RCT | Dutch Bone Metastasis Scoring System | D | 342 | Radiotherapy institutes (n = 17) | Inpatient and outpatient | Palliative radiotherapy | Mixed with bone metastases |
| Wang et al. 2020a (87) | China | Retrospective cohort study | Gastric Cancer Palliative Chemotherapy Nomogram | D + EV  *(in same study)* | D = 259 IV = 50 EV = 147 | Outpatient oncology department in a hospital (n = 1) | Outpatient | Palliative chemotherapy | Gastric |
| Wang et al. 2020b (65) | China | Retrospective cohort study | Gastrointestinal Cancer Home Hospice Nomogram (non-lab nomogram) | D + IV *(random split sample)* | D = 1214 IV = 404 | Hospice unit (n = 1) | Community/home-based | NOT REPORTED | GI |
| Yamada et al. 2017 (55) | Japan | Prospective cohort study | PS-PPI | D + IV *(resampling)* | 2346 | PCUs (n = 16)  Home-based palliative care services (n = 19)  Hospital-based palliative care services (n = 23) | Inpatient and community/home-based | Palliative chemotherapy, hormone therapy, and/or radiotherapy | Mixed |
| Yang et al. 2021 (129) | South Korea | Prospective cohort study | sPPI | EV | 75 | Home-based hospice care (n = 1) | Community/home-based | NOT REPORTED | Mixed |
| Yap et al. 2018 (88) | Taiwan | Retrospective cohort study | Lung bone metastases nomogram (no specific name given) | D + EV  *(in same study)* | D = 477 EV = 235 | Radiation oncology department (n = 1) | Outpatient | Palliative Radiotherapy | Lung with bone metastases |
| Yoon et al. 2014 (104) | South Korea | Retrospective cohort study | OPS | EV | 104 | PCU (n = 1) | Inpatient | NOT REPORTED | Mixed |
| Yoon et al. 2017 (105) | South Korea | Prospective cohort study | OPS | EV | 243 | PCUs (n = 5) | Inpatient | No ongoing anticancer treatment (palliative or supportive care only) | Mixed |
| Yoon et al. 2023 (66) | Japan,  South Korea, Taiwan | Secondary analysis of prospective cohort study | mOPS-A;  mOPS-B | D + IV *(random split sample)* | D = 1290 IV = 506 | PCUs (n = 38) | Inpatient | NOT REPORTED | Mixed |
| Zhao et al. 2019 (77) | China | Retrospective cohort study | Prognostic Nomogram for Advanced Cancer in Palliative Care (Zhao nomogram) | D + IV  *(non-random split sampling)* | D = 247 IV = 131 | PCU (n = 1) | Inpatient | NOT REPORTED | Mixed |

ANN: Artificial Neural Network; BCI: B12/CRP Index; BMETS: Bone Metastases Ensemble Trees for Survival; BPN: Barretos Prognostic Nomogram; CART: Classification and Regression Tree; ChPS: Chinese Prognostic Scale; CRIS: Cochin Risk Index Score; D: Development; D-PaP: Delirium-Palliative Prognostic Score; EOLCI: End of Life Care Index; EV: External Validation; FPN: Feliu Prognostic Nomogram; GI: Gastrointestinal; GI-LPS: Gastrointestinal Malignancy Laboratory Prognostic Score; GPS: Glasgow Prognostic Score; HCC: Hepatocellular Carcinoma; IMPAC: Imminent Mortality Predictor in Advanced Cancer; IV: Internal Validation; JPOS-PI: Japan Palliative Oncology Study–Prognostic Index; LabBM: Laboratory Bone Metastases; LPS: Laboratory Prognostic Score; MDT: Multidisciplinary Team; mGPS: Modified Glasgow Prognostic Score; mGPS-PS: Modified Glasgow Prognostic Score–Performance Status; mOPS-A: Modified Objective Prognostic Score A; mOPS-B: Modified Objective Prognostic Score B; NCCS: National Cancer Centre Singapore; new-ChPS: New Chinese Prognostic Scale; NRF: Number of Risk Factors; OPI-AC: Objective Prognostic Index for Advanced Cancer; OPPS: Objective Palliative Prognostic Score; OPS: Objective Prognostic Score; P1d: Prediction of 1-day Survival; P3did: Prediction of 3-day impending death; PaP: Palliative Prognostic Score; PCU: Palliative Care Unit; PiPS: Prognosis in Palliative Care Study; PPI: Palliative Prognostic Index; PPS: Palliative Performance Scale; PRAIS: Palliative Radiotherapy and Inflammation Study; PRO-MAC: Prognostic Risk Model for Patients with Advanced Cancer; PS-PPI: Performance Status-based Palliative Prognostic Index; R-LPS: Laboratory Prognostic Score for Respiratory Malignancy; RP: Recursive Partitioning; RRRP: Rapid Response Radiotherapy Program; SCS: Simple Clinical Score; sPPI: Simplified Palliative Prognostic Index; SPS: Survival Prediction Score; TEACHH: Type of cancer, ECOG Performance Status, Age, prior palliative Chemotherapy, prior Hospitalisations, and Hepatic metastases; TRIPOD: Transparent reporting of a multivariable prediction model for individual
