# Supplementary material for: End-of-life prognostic models in advanced cancer: a scoping review of model development, validation, and impact
Source: BMC Palliat Care. 2026 Jul 3;25:280. doi: 10.1186/s12904-026-02172-3 (PMC13599129; doi:10.1186/s12904-026-02172-3)
Supplement: Supplementary file 3 [file 12904_2026_2172_MOESM3_ESM.docx]

**Additional File 3.** Characteristics of all identified end-of-life prediction models (n = 77 models across 106 studies).

| **Model Name** | **Clinical Predictors** | **Laboratory Predictors** | **Type of Prediction** | **Time Horizon** | **Intended Population** | **External Validation ^a^** |
| --- | --- | --- | --- | --- | --- | --- |
| 2-Month Prognostic Score (Barbot Score) (28) | KPS (3 classes), number of metastatic sites, dyspnoea, anorexia | Serum albumin, LDH, leukocyte count | Score 0–10; 3 risk groups (A/B/C) | 2-month and 4-month survival | Advanced solid tumour inpatients; estimated survival <6 months | No |
| 7- and 30-Day Survival CART Model (51) | Anorexia, asthenia, level of consciousness, dysphagia, pain, breathlessness, anxiety | None | Probability of death at 7 or 30 days (8 decision tree nodes; range 2–97%) | 7-day and 30-day mortality | Advanced cancer patients visiting palliative care home teams | No |
| ANN 30-Day Survival Model (45) | PPS, KPS, ESAS, presence of metastases, number of comorbidities | Leucocytes, neutrophil count, MCHC, CRP, albumin | Binary classification: alive vs. dead within 30 days | 30-day mortality | Cancer patients admitted to a palliative care unit | No |
| Barnholtz-Sloan WBRT Nomogram (88) | Primary tumour site/histology, status of primary disease, extent of metastatic spread (brain only vs extracranial) | None | Total points (31–212); predicted median, 6-month, and 12-month survival | Median overall survival; 6-month and 12-month survival from WBRT | Advanced cancer patients with brain metastases receiving palliative WBRT | Yes – Norway; France |
| BCI (32, 87) | None (laboratory-only) | Serum vitamin B12, CRP | 3 risk groups (<10,000; 10,001–40,000; >40,000) | 3-month mortality; overall survival | Advanced cancer patients; palliative care unit admission | Yes – UK |
| BMETS (89) | Cancer type, KPS, inpatient status, weight loss (6 months), steroid use, opioid use, prior RT/surgery, RT target site, metastasis count | WBC count, lymphocyte count | Individual predicted survival curves (1, 3, 6, 12 months) | Overall survival at 1, 3, 6, 12 months | Advanced cancer patients with symptomatic bone metastases referred for palliative RT | Yes – USA |
| BPN (74) | Sex, presence of distant metastasis, KPS | WBC count, serum albumin | Survival probability at 30, 90, and 180 days | 30-day, 90-day, 180-day survival | Advanced incurable solid cancer; ambulatory outpatients at first palliative care referral | No |
| BPN v2.0 (75) | Sex, locoregional disease, sites of metastasis | WBC count, serum albumin | Survival probability at 30, 90, and 180 days; worst/typical/best-case scenarios | 30, 90, and 180 days | Outpatients with advanced incurable cancer referred to palliative care | No |
| BPN vClin (75) | Sex, locoregional disease, sites of metastasis | None | Survival probability at 30, 90, and 180 days; worst/typical/best-case scenarios | 30, 90, and 180 days | Outpatients with advanced incurable cancer referred to palliative care | No |
| BPS (111) | ECOG PS and oedema | Cholinesterase, BUN, WBC, CRP, NLR, PLR | Continuous score; ROC-derived cutoff for 3-week survival; combined risk stratification with PPI | 3-week survival | Inpatients with terminal gastrointestinal cancer receiving palliative care | Yes - Japan |
| Chinese Prognostic Prediction Score (69) | KPS, cognitive impairment, severely reduced oral intake, dyspnoea at rest, oedema | WBC, urea, ALT | Score 0–12; binary classification (low <4 vs high ≥4) | 30-day survival | Terminally ill advanced cancer inpatients receiving hospice care | No |
| ChPS (60) | Weight loss, nausea, dysphagia, dyspnoea, oedema, cachexia, dehydration, gender, KPS, QOL score | None | Continuous score 0–124; binary (<3 months vs 3–6 months survival) | 3-month survival; overall survival | Advanced cancer patients in palliative home care; survival <6 months | No |
| Chuang's Prognostic Scale (68, 90, 91) | Lung metastasis, liver metastasis, tiredness, ascites, oedema, cognitive impairment, weight loss, ECOG PS | None | Score 0–8.5; binary survival prediction at cut points | 1-week and 2-week survival (development); 3-month survival (validation studies) | Advanced cancer inpatients; palliative care unit; also validated in metastatic CRC on PSAT | Yes – Saudi Arabia; Egypt |
| Clinical Prediction Model for 1-Year Mortality (3 versions: Simple, Clinical, Extended) (145) | Surprise question, age, cancer type prognosis (good vs intermediate/poor) | Haemoglobin, CRP, serum albumin | Individualised probability of death within 1 year | 1-year mortality | Advanced/metastatic cancer patients receiving palliative-intent treatment | No |
| Combined Initial PPI and Δscore (PPI Change Model) (92) | PPI at admission + change in PPI score over time (initial PPI + Δscore direction) | None | Risk group classification based on initial score + change direction | 30-day mortality; overall survival | Terminally ill cancer patients referred to palliative care; estimated survival <6 months | Yes – Taiwan |
| CRIS (57) | KPS, male sex | Urea, leucocytes, transthyretin | Score; binary risk group (CRIS ≥7 = high risk) | 2-week survival | Terminally ill advanced cancer patients in palliative care unit; no active treatment | No |
| Diagnostic Model for Impending Death within 3 Days (78) | PPS, drooping of nasolabial folds | None | Probability of death at 3-days (4 decision tree nodes; range 3–94%) | 3-day mortality | Advanced cancer patients admitted to acute PCUs | No |
| D-PaP (53) | Dyspnoea, anorexia, KPS, CPS, delirium | Total WBC, lymphocyte percentage | 3 risk groups; 30-day survival probability | 30-day survival | Terminally ill cancer patients in palliative care units | No |
| Dutch Bone Metastasis Scoring System (44, 93) | KPS, primary tumour type (breast/prostate/lung/other), visceral metastases | None | 3 risk groups (A=0–3; B=4–5; C=6 points) | Overall survival (median 7 months) | Advanced cancer patients with painful spinal metastases receiving palliative RT | Yes – Canada |
| Epic EOLCI (94) | Demographics, comorbidities, medications (vendor EHR system; not fully public) | Lab values from EHR (not fully specified); Stanford model includes albumin, anion gap, granulocytes | Score 0–100; risk groups (low/medium/high); 1-year mortality probability | 1-year and 180-day mortality | Outpatient metastatic cancer patients in medical oncology clinics | Yes – USA |
| FPN (80, 106) | ECOG PS, TTD | Albumin, LDH, lymphocyte count | Predicted survival probability at 15, 30, and 60 days | 15-day, 30-day, 60-day survival | Terminally ill cancer patients; progressive malignancy; no life-prolonging therapy | Yes – Spain; UK |
| Gastric Cancer Nomogram (49) | Age, tumour differentiation, ECOG PS, liver metastases, lung metastases, peritoneal metastases | WBC, platelet count, serum albumin, serum bilirubin, ALP | 1-year survival probability; risk classification | 1-year survival | Adults with unresectable/metastatic gastric adenocarcinoma; first-line palliative chemotherapy | No |
| Gastric Cancer Palliative Chemotherapy Nomogram (85) | Number of distant metastatic sites | Fibrinogen, CA199 | 1-year overall survival probability | 1-year overall survival | Advanced/metastatic gastric cancer; good ECOG PS; receiving palliative chemotherapy | Yes – China |
| Gastrointestinal Cancer Home Hospice Nomogram (non-lab nomogram) (65) | KPS, abdominal distension, oedema, QOL score, pain duration | None | 30-day and 60-day survival probability | 30-day and 60-day survival | Advanced gastrointestinal cancer patients in home hospice care; survival <6 months | No |
| GI-LPS (61) | None (laboratory-only) | Total bilirubin, BUN, eosinophil, NLR, RDW | Score 0–5; 3 risk groups; binary cutoff (≥3) for 14-day mortality | 14-day and 30-day mortality | Terminally ill gastrointestinal malignancy patients in hospice | No |
| GPS (95) | ECOG PS, liver metastasis, dyspnoea, oedema | CRP, albumin, NLR | GPS score (0–2); binary risk groups for 3-week and 6-week prognosis | 3-week and 6-week prognosis | Metastatic or locally advanced cancer referred to palliative care | Yes – Japan |
| Graphic tool to estimate individualised survival curves for advanced cancer patients in hospice care (46) | Heart rate, jaundice, ECOG PS, aminoglycoside use, antifungal use | Not specified (Cox PH model variables) | Covariate-adjusted individual survival probability curves over 60 days (with 95%/99% CI) | Continuous survival probability (up to 60 days) | Advanced cancer patients admitted to a hospice ward | No |
| IMPAC (56) | Age, ED entry, prior hospitalisation within 90 days, head/neck or neurological cancer diagnosis | Vital signs, nursing assessments, lab results (via Rothman Index – 26 EHR elements) | Probability of death at 30/60/90/180 days from admission | 30-day, 60-day, 90-day, 180-day mortality | Hospitalised advanced solid tumour patients; ≥48 hours EHR monitoring | No |
| JPOS-PI (81) | CPS, consciousness, pleural effusion | WBC, lymphocyte percentage | Risk group classification (low/intermediate/high) | 30-day survival; overall survival | Advanced solid cancer inpatients; no active cancer-directed therapy | Yes – Japan |
| Kim Prognostic Model (unnamed) (34) | ECOG PS, undergoing chemotherapy (yes/no), anorexia, mid-arm circumference | Lymphocyte percentage, LDH | Score 0–8; binary classification (≥4.0 vs <4.0) | 3-month survival | Advanced cancer patients; oncologist-estimated survival <1 year; outpatient oncology clinic | No |
| Kripp et al. model (35) | ECOG PS, opioid use WHO level, need for blood transfusion | Platelet count, LDH, albumin | Score 0–5; 3 risk groups (low/intermediate/high) | Overall survival; remaining lifespan (days to months) | Adults with advanced haematological malignancies admitted to palliative care unit | Yes – Japan |
| LabBM Score (96) | None (laboratory-only) | CRP >ULN, LDH >ULN, haemoglobin | Score 0–3.5; 3 risk groups (favourable/intermediate/poor) | Overall survival from first day of radiotherapy | Advanced cancer patients receiving palliative radiotherapy (excluding brain metastases) | Yes – Norway |
| Laboratory-free nomogram (59) | QOL scale, KPS, gender, pain duration, anorexia, abdominal distention, fatigue, bone metastasis, prior treatment history | None (laboratory-free) | Survival probability at 30, 45, and 60 days | 30-day, 45-day, 60-day survival | Advanced cancer patients receiving hospice care; labs unavailable or infeasible | No |
| LPS (70) | None (laboratory-only) | CRP, albumin, BUN, WBC, eosinophil, NLR | Score 0–10; cutoff ≥5 for 30-day mortality | 30-day mortality | Terminally ill cancer patients admitted to hospice; no anticancer therapy | No |
| Lung bone metastases nomogram (no specific name given) (86) | Age, histology (NSCLC vs SCLC), smoking status, EGFR mutation status, BMI | NLR | Survival probability at 3, 6, and 12 months | 3-month, 6-month, 12-month survival | Advanced lung cancer patients referred for palliative RT for bone metastases | Yes – Taiwan |
| MDT-adapted PaP (97) | Multidisciplinary team prediction of survival (replaces single clinician CPS), KPS, anorexia, dyspnoea | Total WBC, lymphocyte percentage | 3 risk groups; 30-day survival probability | 30-day survival | Advanced cancer patients newly referred to palliative care | Yes – Australia |
| mGPS (98, 99) | None (biomarker-only) | CRP, serum albumin | Score 0–2; risk group classification | 2-week, 4-week survival; overall survival | Advanced cancer patients near end of life; inpatient and palliative care settings | Yes – UK; Australia |
| mGPS-PS (82) | ECOG PS | CRP and albumin (to calculate mGPS) | 3-month survival probability; risk group classification | 3-month survival; overall survival | Advanced cancer patients on strong opioids / metastatic or locally advanced disease | Yes – UK |
| mOPS-A (66, 146) | Reduced oral intake, dyspnoea at rest, ECOG PS, leg oedema | Leucocytosis, bilirubin, LDH | Score; binary classification (≥2.5 vs <2.5) | 2-week survival (primary); 3-week (secondary) | Advanced cancer inpatients in palliative care units | Yes – Taiwan |
| mOPS-B (66, 146) | Reduced oral intake, dyspnoea at rest, ECOG PS, leg oedema, drowsiness (IPOS) | None (laboratory-free) | Score 0–4; binary classification (≥1.5 high risk) | 2-week survival | Advanced cancer inpatients in palliative care units; feasible when labs unavailable | Yes – Taiwan |
| NCCS Prognostic Model (63) | Inpatient status at start of RT, PPS, prior chemotherapy within 3 months | NLR | Survival probability estimate; 3 risk groups (0/1/2–3 risk factors) | Overall survival from start of RT | Advanced cancer patients receiving palliative-intent external beam radiotherapy | No |
| new-ChPS (31) | KPS, pain, ascites, hydrothorax, oedema, delirium, cachexia | WBC, haemoglobin, sodium, total bilirubin, direct bilirubin, AST, ALP | Score 0–30; 5 risk groups; cutoff-based survival prediction | 7-day, 30-day, 90-day, 180-day survival | Hospitalised advanced cancer patients; expected to die within 6 months | No |
| OPI-AC (48) | None (objective variables: heart rate, respiratory rate) | Heart rate, respiratory rate, urea, albumin, CRP, creatinine, lymphocyte count, LDH, platelet count | Predicted survival probability at 7, 14, 30, 56, and 90 days | 7-day, 14-day, 30-day, 56-day, 90-day survival | Advanced cancer patients (including haematological) receiving specialist palliative care | No |
| OPPS (29) | History of prior chemotherapy, heart rate | WBC, platelets, creatinine, potassium | Score 0–6; binary groups (0–2 vs 3 vs 4–5) for 7-day mortality probability | 7-day mortality | Advanced cancer patients admitted to a hospice palliative care unit | No |
| OPS (43, 101-104) | Reduced oral intake (anorexia), resting dyspnoea, ECOG PS | WBC, serum bilirubin, serum creatinine, serum LDH | Score 0–7; binary classification (OPS ≥3 vs <3) | 3-week mortality | Terminally ill inpatient advanced cancer; no anticancer treatment | Yes – Italy; Japan; South Korea |
| OPS (Okugawa variant) (73) | None (laboratory-only) | ALT, total bilirubin, BUN, creatinine, platelet count | Continuous probability score 0–1; binary classification at cutoff 0.735 | Death within 1 week; overall survival | Advanced cancer patients admitted to palliative care unit | No |
| P1d-Survival Decision Tree (38) | Response to verbal stimuli, peripheral cyanosis, mandibular movement, Cheyne-Stokes breathing, apnoeic periods, death rattle | None (bedside clinical signs only) | Risk groups with next-day survival probability (%) | Next-day (1-day) survival | Advanced cancer patients with PPS ≤20; imminently dying | No |
| P3did-Decision Tree (79) | Urine output, decreased response to verbal stimuli, RASS score | None | Probability of death at 3-days (4 decision tree nodes; range 20.6–80.3%) | 3-day mortality | Advanced cancer patients with PPS ≤20; imminently dying | No |
| P3did Score (79) | Signs across 4 systems: nervous (RASS score), cardiovascular (peripheral cyanosis, pulselessness of radial artery, decreased urine output), respiratory (apnoea, Cheyne-Stokes breathing, respiration with mandibular movement), musculoskeletal (inability to close eyelids, hyperextension of neck, drooping of nasolabial folds) | None | Score 0–4; 3-day mortality probability per score point | 3-day mortality | Advanced cancer patients with PPS ≤20; imminently dying | No |
| PaP (40, 104, 106, 114) (97, 105, 119) | Dyspnoea, anorexia, KPS, CPS | Total WBC count, lymphocyte percentage | Continuous score (0–17.5); 3 risk groups; 30-day survival probability | 30-day survival | Advanced solid tumour patients; antiblastic therapy no longer viable | Yes – Italy; South Korea; Japan |
| PaP Score Nomogram (83) | Dyspnoea, anorexia, KPS, CPS | Total WBC, lymphocyte percentage | Individualised survival probability at 15, 30, and 60 days | 15-day, 30-day, 60-day survival | Terminally ill advanced solid tumour patients; antiblastic therapy no longer viable | Yes – Italy |
| PiPS-A (47, 106) | ECOG PS, global health status, pulse rate, anorexia, metastasis site, primary cancer site, cognitive impairment | None | Categorical: "days" (<14 days), "weeks" (14–55 days), "months+" (>55 days) | 14-day and 56-day survival | Advanced cancer patients no longer receiving disease-modifying treatment | Yes – UK |
| PiPS-B (47, 106) | ECOG PS, global health status, pulse rate, anorexia, metastasis site, primary cancer site, cognitive impairment | CRP, WBC, platelet count, urea, ALT, neutrophil count, lymphocyte count, ALP, LDH, sodium, albumin | Categorical: "days" (<14 days), "weeks" (14–55 days), "months+" (>55 days) | 14-day and 56-day survival | Advanced cancer patients no longer receiving disease-modifying treatment | Yes – UK |
| PPI (72, 104, 106-119, 130) | PPS, oral intake, oedema, dyspnoea at rest, delirium | None | Continuous score (0–15); 3 risk groups (A/B/C) | 3-week and 6-week survival | Advanced cancer patients in palliative/hospice care | Yes – Kuwait; Brazil, Taiwan; Italy; Japan; South Korea; Indonesia; Republic of Ireland; UK; India |
| PPS-based 90-Day Mortality Model (120) | PPS, gender, prior oncologic treatment within 3 months | Albumin, WBC, haemoglobin, platelet count, calcium | Probability of 90-day mortality; binary risk classification | 90-day mortality; overall survival | Advanced cancer patients admitted to palliative care unit | Yes – Singapore |
| PRAIS predictive models (52) | Primary tumour site, non-bone metastases, opioid intake, steroid intake, food intake, BMI | IL-8, serum chloride, CRP, WBC, lymphocyte count | Predicted survival probability at 3 weeks, 24 weeks, and 52 weeks | 3 weeks, 24 weeks, 52 weeks | Advanced cancer patients with bone metastases referred for palliative radiotherapy | No |
| Prognostic Index for Advanced Pancreatic Cancer (50) | ECOG PS, distant metastatic disease, status of initially unresectable disease | CEA, CA19-9, NLR | Score 0–8; 3 risk groups (favourable/intermediate/poor) | 1-year survival; overall survival | Adults with unresectable or recurrent pancreatic ductal adenocarcinoma; first-line palliative chemotherapy | No |
| Prognostic model for survival prediction for patients with advanced cancer in a palliative care setting (71) | ECOG PS, general fatigue (patient-reported) | Albumin, LDH, lymphocyte percentage | Score 0–7.5; cutoff >4.5 for 60-day survival | 30-day and 60-day survival | Advanced cancer patients admitted to palliative care unit | No |
| Prognostic model using routine laboratory results (37) | None | Total bilirubin, AST, creatinine, urea/creatinine ratio, eosinophil percentage, lymphocyte count | Score 0–14; 3 risk groups (low/medium/high) | Overall survival (up to 180 days) | Advanced cancer patients admitted to palliative care; no active treatment | No |
| Prognostic Nomogram for Advanced Cancer in Palliative Care (Zhao nomogram) (77) | Sex, age, TNM tumour stage, KPS | Neutrophil count, haemoglobin, LDH, albumin, uric acid, cystatin-C | 90-day mortality probability (continuous nomogram) | 90-day mortality; overall survival | Advanced cancer patients in palliative care | No |
| Prognostic scale for 7-day survival prediction (67) | Cognitive status, oedema, ECOG PS, respiratory rate | BUN | Continuous probability of dying within 7 days; binary at cutoff >0.2 | 7-day mortality | Terminal cancer patients admitted to inpatient palliative care/hospice ward | No |
| PRO-MAC (58) | Palliative Performance Scale v2, ESAS-r summative score, number of organ systems affected by metastases | Serum albumin, total WBC | Score 0–10.5; 3 risk groups (low/medium/high) | 30-day and 90-day mortality | Advanced cancer patients admitted or referred to palliative care | No |
| PRONOPALL Score (121) | ECOG PS, number of metastatic sites | LDH, serum albumin | Score 0–10; 3 risk groups (A/B/C) | 2-month and 6-month survival; overall survival | Advanced incurable cancer patients at palliative stage; receiving anticancer treatment | Yes – France |
| PS-PPI (55) | ECOG PS (replaces PPS), oral intake, oedema, dyspnoea at rest, delirium | None | Score 0–15; 3 risk groups (low/medium/high) | 3 weeks, 6 weeks, 30–180 days | Palliative care patients; validated in haematological malignancies and mixed tumour types | No |
| Rades et al. three-tiered survival score (41, 122) | KPS, N stage, M stage | None | Point sum (10–17); 3 risk groups | 3-month, 6-month, 12-month survival | Adults with locally advanced lung cancer; palliative radiotherapy; unable to tolerate curative treatment | Yes – Norway |
| Recursive Partitioning (RP) Prognostic Model (79) | KPS, site of metastases | None | 3 risk groups | Survival at 3, 6, and 12 months; median survival | Advanced cancer patients referred for palliative radiotherapy | Yes – Canada |
| Risk Scoring System for 6-Month Mortality in Gastric Cancer with Peritoneal Carcinomatosis (39) | Age, female sex, ASA III/IV, Charlson Comorbidity Index, ascites on CT, stenosis on CT, tumour size | Haemoglobin, NLR | Score 0–17; binary risk classification (cutoff 9 points) | 6-month mortality | Adults with gastric cancer and peritoneal carcinomatosis; clinical stage IV | No |
| R-LPS (64) | None (laboratory-only) | CRP, AST, BUN, WBC, eosinophil, NLR, RDW, platelets | Integer score 0–8; risk group classification | 14-day mortality; 30-day survival | Terminally ill respiratory malignancy patients in palliative care unit | No |
| RRRP 3-variable NRF/SPS Model (78, 118, 123-125, 129) | Primary cancer site (breast/prostate vs lung/other), site of metastases (bone only vs other), KPS | None (simplified; ESAS symptom variables removed) | 3 risk groups; survival probability at 3, 6, and 12 months | 3-month, 6-month, 12-month survival | Advanced cancer patients referred for palliative radiotherapy | Yes – Brazil; Canada; USA; Japan |
| RRRP 6-variable NRF/SPS Model (30, 93, 126) | Primary cancer site, site of metastases, KPS, ESAS fatigue score, ESAS appetite score, ESAS shortness of breath score | None | 3 risk groups; survival probability at 3, 6, and 12 months | 3-month, 6-month, 12-month survival | Advanced cancer patients referred for palliative radiotherapy | Yes – Canada |
| Schonwetter Terminal Lung Cancer Prognostic Model (76) | Pulse rate, toileting capacity, feeding capacity, living situation, quality of sleep | None | Score 0–8; survival time estimates at 50th and 90th percentiles | Short-term survival (days); median survival by score group | Terminal lung cancer patients in hospice | No |
| SCS (42) | Cancer type (pancreatic/ampullary/cholangiocarcinoma), presence of liver metastasis | None | Binary classification: SCS <0 (survival ≥24 weeks) vs SCS ≥0 (<24 weeks) | Survival <24 weeks (6 months) vs ≥24 weeks | Adults with inoperable malignant distal biliary obstruction; palliative endoscopic biliary drainage | No |
| sPPI (33, 127) | PPS, oral intake, oedema, dyspnoea at rest, Communication Capacity Scale Item 4 (substitutes for delirium) | None | Continuous score; risk groups (A/B/C) | 3-week and 6-week survival | Palliative care unit patients including haematological malignancies | Yes – South Korea |
| Stone Prognostic Index (unnamed) (54) | Primary lung cancer, secondary liver cancer, ECOG PS | CRP | Binary risk groups (low risk: score <2; high risk: score ≥2) | 14-day survival; overall survival | Advanced cancer; no disease-modifying treatment; multiple care settings | No |
| Survival score for elderly patients (≥65 years) with bone metastases receiving radiotherapy (unnamed) (62) | Gender, ECOG PS, primary tumour type, site(s) of metastases, interval diagnosis to RT | None | 3 risk groups (A/B/C); 6-month and 12-month survival rates | 6 months, 12 months | Elderly adults (≥65 years) with bone metastases receiving palliative radiotherapy | No |
| TEACHH Model (36, 128, 129) | Cancer type (breast/prostate vs lung vs other), ECOG PS, age, number of prior palliative chemotherapy courses, prior hospitalisation within 3 months, hepatic metastases | None | 3 risk groups (A/B/C) with median life expectancy estimates | <3 months and >1 year survival | Advanced cancer patients referred for palliative radiotherapy | Yes – Canada; USA |
| Tournoux-Facon HCC Palliative Score (84) | Tumour morphology (Milan criteria), portal vein obstruction, metastasis, WHO PS, ascites, jaundice | AFP, ALP >2x ULN | 3 risk groups (low/intermediate/high) | Overall survival (~4–7 months median) | Advanced HCC in palliative setting; BCLC stage C/D; ineligible for curative treatment | Yes – France |

AFP: alpha-fetoprotein; ALP: alkaline phosphatase; ALT: alanine aminotransferase; ANN: Artificial Neural Network; AST: aspartate aminotransferase; BCLC: Barcelona Clinic Liver Cancer staging; BCI: B12/CRP Index; BMETS: Bone Metastases Ensemble Trees for Survival; BMI: body mass index; BPN: Barretos Prognostic Nomogram; BPS: Biological Prognostic Score; BUN: blood urea nitrogen; CA19-9: carbohydrate antigen 19-9; CART: Classification and Regression Tree; CEA: carcinoembryonic antigen; ChPS: Chinese Prognostic Scale; CNS: central nervous system; CPS: clinical prediction of survival; CRC: colorectal cancer; CRIS: Cochin Risk Index Score; CRP: C-reactive protein; CT: computed tomography; D-PaP: Delirium-Palliative Prognostic Score; ECOG PS: Eastern Cooperative Oncology Group Performance Status; EHR: electronic health record; EGFR: epidermal growth factor receptor; EOLCI: End of Life Care Index; ESAS: Edmonton Symptom Assessment System; ESAS-r: Edmonton Symptom Assessment System revised; EV: external validation; FPN: Feliu Prognostic Nomogram; GI: Gastrointestinal; GI-LPS: Gastrointestinal Malignancy Laboratory Prognostic Score; GPS: Glasgow Prognostic Score; HAprog: refined Barretos Prognostic Nomogram; HCC: Hepatocellular Carcinoma; IL-8: interleukin-8; IMPAC: Imminent Mortality Predictor in Advanced Cancer; IPOS: Integrated Palliative care Outcome Scale; IV: internal validation; JPOS-PI: Japan Palliative Oncology Study–Prognostic Index; KPS: Karnofsky Performance Score; LabBM: Laboratory Bone Metastases; LDH: lactate dehydrogenase; LPS: Laboratory Prognostic Score; MCHC: mean corpuscular haemoglobin concentration; MDT: Multidisciplinary Team; mGPS: Modified Glasgow Prognostic Score; mGPS-PS: Modified Glasgow Prognostic Score–Performance Status; mOPS-A: Modified Objective Prognostic Score A; mOPS-B: Modified Objective Prognostic Score B; MTPS-PaP: Multidisciplinary Team Prediction of Survival-adapted Palliative Prognostic Score; NCCS: National Cancer Centre Singapore; new-ChPS: New Chinese Prognostic Scale; NLR: neutrophil-to-lymphocyte ratio; NSCLC: non-small-cell lung cancer; OPI-AC: Objective Prognostic Index for Advanced Cancer; OPPS: Objective Palliative Prognostic Score; OPS: Objective Prognostic Score; P1d: Prediction of 1-day Survival; P3did: Prediction of 3-day impending death; PaP: Palliative Prognostic Score; PCT: palliative care team; PCU: Palliative Care Unit; PiPS: Prognosis in Palliative Care Study; PiPS-A: Prognosis in Palliative Care Study model A; PiPS-B: Prognosis in Palliative Care Study model B; PLR: platelet-to-lymphocyte ratio; PPI: Palliative Prognostic Index; PPS: Palliative Performance Scale; PRAIS: Palliative Radiotherapy and Inflammation Study; PRO-MAC: Prognostic Risk Model for Patients with Advanced Cancer; PRONOPALL: palliative oncology prognostic score for patients receiving anticancer treatment; PS-PPI: Performance Status-based Palliative Prognostic Index; PSAT: palliative systemic anticancer therapy; QOL: quality of life; R-LPS: Laboratory Prognostic Score for Respiratory Malignancy; RDW: red cell distribution width; RP: Recursive Partitioning; RRRP: Rapid Response Radiotherapy Program; RT: radiotherapy; SCS: Simple Clinical Score; SCLC: small-cell lung cancer; SHHCP-NOMO: laboratory-free nomogram for hospice care; sPPI: Simplified Palliative Prognostic Index; SPS: Survival Prediction Score; TEACHH: Type of cancer, ECOG Performance Status, Age, prior palliative Chemotherapy, prior Hospitalisations, and Hepatic metastases; TNM: tumour-node-metastasis staging; TRIPOD: Transparent reporting of a multivariable prediction model for individual prognosis or diagnosis; TTD: time from initial diagnosis to terminal disease diagnosis; UK: United Kingdom; ULN: upper limit of normal; USA: United States of America; WBRT: Whole-Brain Radiotherapy; WBC: white blood cell count; WHO: World Health Organization.

^a^ Models listed as 'No' external validation were developed and/or internally validated only. 'Yes' indicates validation in at least one independent external dataset; countries listed reflect those explicitly stated in included study reports.
