# Supplementary material for: End-of-life prognostic models in advanced cancer: a scoping review of model development, validation, and impact
Source: BMC Palliat Care. 2026 Jul 3;25:280. doi: 10.1186/s12904-026-02172-3 (PMC13599129; doi:10.1186/s12904-026-02172-3)
Supplement: Supplementary file 4 [file 12904_2026_2172_MOESM4_ESM.docx]

**Additional File 4.** Summary of externally validated end-of-life prediction models

| **Model** | **Study (Author, Year, Ref)** | **Country of Validation** | **Cancer Population** | **Clinical Setting(s)** | **Setting Type** | **Treatment Status** |
| --- | --- | --- | --- | --- | --- | --- |
| PPI | Alshemmari et al. 2012 (107) | Kuwait | Mixed advanced cancers | Cancer hospital (n=1) | Inpatient | No ongoing anticancer treatment |
|  | Cheng et al. 2012 (108) | Taiwan | Mixed advanced cancers | Hospice consultation service, medical centre (n=1) | Inpatient | No ongoing anticancer treatment |
|  | Kim et al. 2014 (119) | South Korea | Mixed terminal cancers | Hospices (n=2) | Inpatient | No ongoing anticancer treatment |
|  | Sonoda et al. 2014 (114) | Japan | Mixed advanced cancers | Palliative care consultation service, hospital (n=1) | Inpatient | No ongoing anticancer treatment |
|  | Inomata et al. 2014 (110) | Japan | Lung cancer | Hospital (n=1) | Inpatient | No ongoing anticancer treatment |
|  | Stone et al. 2008a (130) | Republic of Ireland | Mixed advanced cancers | Specialist palliative care service, hospital (n=1) | Inpatient, outpatient, and community | Palliative chemotherapy and/or radiotherapy |
|  | Subramaniam et al. 2013 (115) | UK | Mixed advanced cancers | Hospices (n=3) | Inpatient | NOT REPORTED |
|  | Ohno et al. 2017 (112) | Japan | Advanced haematological malignancies | Haematology ward, hospital (n=1) | Inpatient | Palliative chemotherapy and/or radiotherapy |
|  | Chen et al. 2018 (118) | Brazil | Mixed advanced cancers | Cancer centre (n=1) | Inpatient | Palliative radiotherapy |
|  | Ermacora et al. 2019 (104) | Italy | Mixed advanced solid tumours | Palliative care units (n=2) | Inpatient | No ongoing anticancer treatment |
|  | Kadokura et al. 2020 (111) | Japan | Terminal gastrointestinal cancers | Hospital (n=1) | Inpatient | Non-curative (palliative) |
|  | Subramaniam et al. 2019 (116) | UK | Mixed advanced cancers | Hospices (n=10) | Inpatient | NOT REPORTED |
|  | Shatri et al. 2021 (113) | Indonesia | Mixed advanced cancers | Tertiary referral hospital (n=1) | Inpatient | NOT REPORTED |
|  | Stone et al. 2021 (106) | UK | Mixed advanced incurable cancers | Multiple palliative care services (n=27) | Inpatient, outpatient, and community | No ongoing anticancer treatment |
|  | Iizuka-Honma et al. 2022 (109) | Japan | Haematological malignancies | Haematology ward, hospital (n=1) | Inpatient | Palliative chemotherapy |
|  | Tiwari et al. 2024 (117) | India | Mixed advanced cancers | Palliative care clinic (n=1) | Outpatient | Palliative chemotherapy and/or radiotherapy |
| RRRP 3-variable NRF/SPS Model | Chow et al. 2008 (78) | Canada | Mixed metastatic cancers | Outpatient palliative radiotherapy clinics (n=2) | Outpatient | Palliative radiotherapy |
|  | Glare et al. 2014 (124) | USA | Mixed advanced cancers | Pain and palliative care outpatient clinic (n=1) | Outpatient | No ongoing anticancer treatment |
|  | Chow et al. 2011 (123) | USA; Canada | Breast and prostate cancer with bone metastases | Palliative radiation facilities (n=66) | Outpatient | Palliative radiotherapy |
|  | Chen et al. 2018 (118) | Brazil | Mixed advanced cancers | Cancer centre (n=1) | Inpatient | Palliative radiotherapy |
|  | Mojica-Marquez et al. 2020 (129) | USA | Mixed advanced cancers | Integrated cancer network (academic and community) | Inpatient/outpatient | Palliative radiotherapy |
|  | Sakurai et al. 2024 (125) | Japan | Mixed cancers with bone and non-bone metastases | Radiation oncology departments (n=4) | NR | Palliative radiotherapy |
| PaP | Maltoni et al. 1999 (105) | Italy | Mixed advanced solid tumours | Palliative care centres (n=14) | Inpatient, outpatient, and home | Palliative steroid and/or hormone therapy |
|  | Kim et al. 2014 (119) | South Korea | Mixed terminal cancers | Hospices (n=2) | Inpatient | No ongoing anticancer treatment |
|  | Ermacora et al. 2019 (104) | Italy | Mixed advanced solid tumours | Palliative care units (n=2) | Inpatient | No ongoing anticancer treatment |
|  | Sonoda et al. 2014 (114) | Japan | Mixed advanced cancers | Palliative care consultation service, hospital (n=1) | Inpatient | No ongoing anticancer treatment |
|  | Stone et al. 2021 (106) | UK | Mixed advanced incurable cancers | Multiple palliative care services (n=27) | Inpatient, outpatient, and community | No ongoing anticancer treatment |
| OPS | Ermacora et al. 2019 (104) | Italy | Mixed advanced solid tumours | Palliative care units (n=2) | Inpatient | No ongoing anticancer treatment |
|  | Jho et al. 2016 (101) | Japan | Mixed advanced cancers | Palliative care teams, PCUs, home-based care | Inpatient, outpatient, and community | No ongoing anticancer treatment |
|  | Yoon et al. 2014 (102) | South Korea | Mixed terminal cancers | Palliative care unit (n=1) | Inpatient | NOT REPORTED |
|  | Yoon et al. 2017 (103) | South Korea | Mixed advanced cancers | Palliative care units (n=5) | Inpatient | No ongoing anticancer treatment |
| Chuang's Prognostic Scale | Alsirafy et al. 2016 (90) | Egypt | Metastatic colorectal cancer on PSAT | Outpatient oncology clinic (n=1) | Outpatient | Palliative systemic anticancer therapy |
|  | Al-Zahrani et al. 2012 (91) | Saudi Arabia | Mixed advanced cancers | Inpatient oncology ward, hospital (n=1) | Inpatient | No ongoing anticancer treatment |
| FPN | Feliu et al. 2011 (80) | Spain | Mixed advanced cancers | Oncology and palliative care units (n=11) | Inpatient and outpatient | No ongoing anticancer treatment |
|  | Stone et al. 2021 (106) | UK | Mixed advanced incurable cancers | Multiple palliative care services (n=27) | Inpatient, outpatient, and community | No ongoing anticancer treatment |
| mGPS | Partridge et al. 2012 (99) | UK | Mixed advanced cancers near end of life | Inpatient hospice (n=1) | Inpatient | NOT REPORTED |
|  | Lojanapiwat et al. 2021 (98) | Australia | Mixed advanced cancers | Acute regional hospital (n=1) | Inpatient | NOT REPORTED |
| mOPS-A | Hiratsuka et al. 2024 (100) | Taiwan | Mixed advanced cancers | Palliative care units (n=4) | Inpatient | No ongoing anticancer treatment |
| mOPS-B | Hiratsuka et al. 2024 (100) | Taiwan | Mixed advanced cancers | Palliative care units (n=4) | Inpatient | No ongoing anticancer treatment |
| PiPS-A | Stone et al. 2021 (106) | UK | Mixed advanced incurable cancers | Multiple palliative care services (n=27) | Inpatient, outpatient, and community | No ongoing anticancer treatment |
| PiPS-B | Stone et al. 2021 (106) | UK | Mixed advanced incurable cancers | Multiple palliative care services (n=27) | Inpatient, outpatient, and community | No ongoing anticancer treatment |
| RRRP 6-variable SPS/NRF Model | Chow et al. 2006 (93) | Canada | Mixed cancers with spinal bone metastases | Outpatient palliative radiotherapy clinic (n=1) | Outpatient | Palliative radiotherapy |
|  | Chow et al. 2009a (126) | Canada | Mixed metastatic cancers | Outpatient palliative radiotherapy clinic (n=1) | Outpatient | Palliative radiotherapy |
| TEACHH Model | Dosani et al. 2018 (128) | Canada | Mixed cancers with spinal metastases | Population-based cancer centre (n=1) | Outpatient | Palliative radiotherapy |
|  | Mojica-Marquez et al. 2020 (129) | USA | Mixed advanced cancers | Integrated cancer network (academic and community) | Inpatient/outpatient | Palliative radiotherapy |
| Barnholtz-Sloan WBRT Nomogram | Nieder et al. 2017a (88) | Norway; Germany | Mixed solid tumours with brain metastases | Radiotherapy departments (n=2) | Inpatient and outpatient | Palliative whole brain radiotherapy |
| BCI | Kelly et al. 2007 (87) | UK | Mixed advanced cancers | Hospitals (n=3) and hospices (n=4) | Inpatient and outpatient | No ongoing anticancer treatment |
| BMETS | Elledge et al. 2021 (89) | USA | Mixed cancers with symptomatic bone metastases | Community radiation oncology clinics (n=2) | Outpatient | Palliative radiotherapy |
| BPS | Kadokura et al. 2020 (111) | Japan | Terminal gastrointestinal cancers | Hospital (n=1) | Inpatient | Non-curative (palliative) |
| Combined Initial PPI and Δscore (PPI Change Model) | Kao et al. 2014 (92) | Taiwan | Mixed terminal cancers | Palliative care consultation service, hospital (n=1) | Inpatient | No ongoing anticancer treatment |
| Dutch Bone Metastasis Scoring System | Chow et al. 2006 (93) | Canada | Mixed solid tumours with spinal bone metastases | Outpatient palliative radiotherapy clinic (n=1) | Outpatient | Palliative radiotherapy |
| Epic EOLCI | Gensheimer et al. 2025 (94) | USA | Mixed metastatic cancers | Medical oncology outpatient clinics (n=4) | Outpatient | NOT REPORTED |
| Gastric Cancer Palliative Chemotherapy Nomogram | Wang et al. 2020a (85) | China | Unresectable / metastatic gastric cancer | Outpatient oncology department (n=1) | Outpatient | Palliative chemotherapy |
| GPS | Miura et al. 2015 (95) | Japan | Mixed advanced cancers | PCUs (n=16), hospital palliative care teams (n=19), home-based palliative care services (n=23) | Inpatient and home-based | No ongoing anticancer treatment |
| JPOS-PI | Hyodo et al. 2010 (81) | Japan | Mixed advanced solid tumours | Palliative care units (n=14) | Inpatient | No ongoing anticancer treatment |
| Kripp et al. model | Ohno et al. 2017 (112) | Japan | Advanced haematological malignancies | Haematology ward, hospital (n=1) | Inpatient | Palliative chemotherapy and/or radiotherapy |
| LabBM Score | Nieder et al. 2021 (96) | Norway | Mixed advanced cancers (excl. brain metastases and haematological malignancies) | Radiotherapy department, hospital (n=1) | Inpatient and outpatient | Palliative radiotherapy |
| Lung Bone Metastases Nomogram | Yap et al. 2018 (86) | Taiwan | Metastatic lung cancer with bone metastases | Radiation oncology department (n=1) | Outpatient | Palliative radiotherapy |
| MDT-adapted PaP | Mendis et al. 2015 (97) | Australia | Mixed solid malignancies | Palliative care consultation service, tertiary cancer centre (n=1) | Inpatient and outpatient | NOT REPORTED |
| mGPS-PS | Laird et al. 2013 (82) | Denmark; Finland; Germany; UK; Iceland; Italy; Lithuania; Norway; Sweden; Switzerland; Greece | Mixed advanced cancers | Oncology wards, PCUs/hospices, surgical wards, outpatient depts (multi-country) | Inpatient and outpatient | Palliative chemotherapy |
| PaP Score Nomogram | Scarpi et al. 2022 (83) | Italy | Mixed advanced solid tumours | Hospices (multi-centre) | Inpatient | Palliative hormone therapy and/or radiotherapy |
| PPS-based 90-Day Mortality Model | Mei et al. 2013 (120) | Singapore | Mixed advanced cancers | Palliative care unit (n=1) | Inpatient | NOT REPORTED |
| PRONOPALL Score | Bourgeois et al. 2017 (121) | France | Mixed solid tumours receiving palliative anticancer therapy | Oncology centres (n=16) | Outpatient | Palliative anticancer therapy |
| Rades et al. Three-Tiered Survival Score | Nieder et al. 2017b (122) | Norway | Lung cancer (NSCLC and SCLC) | Radiotherapy department, hospital (n=1) | Inpatient and outpatient | Palliative radiotherapy |
| Recursive Partitioning (RP) Prognostic Model | Chow et al. 2009b (79) | Canada | Mixed advanced cancers | Outpatient palliative radiotherapy clinic (n=2) | Outpatient | Palliative radiotherapy |
| sPPI | Yang et al. 2021 (127) | South Korea | Mixed advanced cancers | Home-based hospice care (n=1) | Community / home-based | NOT REPORTED |
| Tournoux-Facon HCC Palliative Score | Tournoux-Facon et al. 2011 (84) | France | Hepatocellular carcinoma, palliative setting | Multi-centre RCT palliative care settings | Mixed (multi-centre) | No ongoing anticancer treatment |

BCI: B12/CRP Index; BMETS: Bone Metastases Ensemble Trees for Survival; BPS: Biological Prognostic Score; CRP: C-reactive protein; ECOG PS: Eastern Cooperative Oncology Group Performance Status; EOLCI: End of Life Care Index; FPN: Feliu Prognostic Nomogram; GPS: Glasgow Prognostic Score; HCC: Hepatocellular carcinoma; JPOS-PI: Japan Palliative Oncology Study – Prognostic Index; LabBM: Laboratory Bone Metastases score; MDT: Multidisciplinary team; mGPS: Modified Glasgow Prognostic Score; mGPS-PS: Modified Glasgow Prognostic Score – Performance Status; mOPS-A: Modified Objective Prognostic Score A; mOPS-B: Modified Objective Prognostic Score B; NRF: Number of Risk Factors; NSCLC: Non-small-cell lung cancer; OPS: Objective Prognostic Score; PaP: Palliative Prognostic Score; PCU: Palliative care unit; PiPS-A: Prognosis in Palliative care Study model A; PiPS-B: Prognosis in Palliative care Study model B; PPI: Palliative Prognostic Index; PPS: Palliative Performance Scale; PRONOPALL: Palliative oncology prognostic score for patients receiving anticancer treatment; PSAT: Palliative systemic anticancer therapy; RCT: Randomised controlled trial; RP: Recursive Partitioning; RRRP: Rapid Response Radiotherapy Program; SCLC: Small-cell lung cancer; sPPI: Simplified Palliative Prognostic Index; SPS: Survival Prediction Score; TEACHH: Type of cancer, ECOG Performance Status, Age, prior palliative CHemotherapy, prior Hospitalisations, and Hepatic metastases; UK: United Kingdom; USA: United States of America; WBRT: Whole brain radiotherapy.
